# Feature stabilization in convolutional neural networks using Proportional Integral Controller for lung nodule classification

**DOI:** 10.3389/frai.2026.1794876

**Published:** 2026-04-30

**Authors:** V. Sangeetha, S. Kalaivani

**Affiliations:** School of Computer Science, Engineering and Information Systems, Vellore Institute of Technology, Vellore, India

**Keywords:** Proportional plus Integral Controller, feature map, convolutional neural network, lung nodules, deep learning, classification

## Abstract

**Introduction:**

Reliable classification of lung nodules from computed tomography (CT) images remains a challenging problem due to variations in image intensity, noise, and unstable feature representations during deep network training. Although convolutional neural networks (CNNs) have achieved promising results in medical image analysis, their internal feature dynamics are often difficult to control, which can affect convergence stability and generalization, particularly when working with limited clinical data.

**Method:**

In this work, we propose a control-inspired CNN framework that incorporates a Proportional Integral Controller (PIC) to regulate feature representations during the learning process. The PIC is integrated into the network in two distinct ways: as a preprocessing module before the CNN and as an intermediate layer embedded within the convolutional architecture. Both manually tuned and automatically learned PIC configurations are investigated to analyze the influence of fixed, knowledge-driven control parameters vs. adaptive, data-driven feedback mechanisms. The proportional component responds to instantaneous feature deviations, while the integral component compensates for accumulated errors, jointly contributing to more stable and consistent feature learning.

**Result:**

The proposed approach is evaluated on the IQ-OTH/NCCD lung cancer dataset using standard classification metrics. The proposed method achieves state-of-the-art performance (Accuracy 0.96, F1-score 0.96) and eliminates false positives (Precision 0.93). Ablation and statistical analyses confirm the importance of PIC placement and parameter tuning, while cross-dataset validation demonstrates strong generalization. Overall, this study demonstrates that integrating principles from control theory into deep learning architectures provides an effective and interpretable strategy for enhancing medical image classification.

## 1 Introduction

Lung cancer is a major cause of cancer-related deaths all over the world, and early diagnosis using pulmonary nodule classification would provide a paramount chance of better patient outcomes (Huang et al., 2021). Convolutional Neural Networks (CNNs) have become the core of computer-aided diagnosis systems, and they have proven to be quite powerful in terms of extracting the features in the computed tomography (CT) images (Liz-López et al., 2025). Nevertheless, although CNN training is essentially challenged by the instability of the feature representation, its hierarchical capabilities of feature learning, with early layers capturing edge and texture information, and deep layers encoding semantic representations (Xu et al., 2023).

It has been found that the phenomenon of internal covariate shift (ICS), where the distribution of layer activations varies continuously in training as the parameters of previous layers change, is a major barrier to stable convergence (Ioffe and Szegedy, 2015). Although Batch Normalization and other models (Layer Normalization, Group Normalization) offer some solutions since they standardize layer outputs, these models are passive and reactive, and normalization distributions are applied instead of error trends being actively monitored, and feedback corrective measures are given in relation to cumulative drift^1^. This weakness is severe in medical imaging, where small datasets have a disproportionate effect on representation drift and training instability on intermediate and deep layers (Farchi et al., 2024).

Recent developments have discussed hybrid architectures that combine the complementary capabilities of CNNs and Vision Transformers (ViTs). ViT-GCN (Xu et al., 2025) combines Vision Transformer and Graph Convolutional Networks to obtain both world image data and relational node data (91.43% accuracy on pneumonia in chest X-rays). Likewise, BiCFormer uses Swin Transformer (Zhao et al., 2024) that uses bi-level coordinate attention to get 97.4% accuracy on benign/malignant pulmonary nodule classification. These architectural inventions enhance the performance of classification by structural hybridization, but are based on passive normalization (LayerNorm, BatchNorm), and do not consider the underlying training dynamics or feature drift that exists within an individual branch during training.

More importantly, the literature does not utilize active feedback control to regularize CNN feature representations during training in lung nodule classification. We consider it a research gap: the lack of an intra-network process of cumulative error correction, among sequential convolutional layers. Although PIDNet has shown that the Proportional-Integral-Derivative (PID) principles of control might be used to mitigate the phenomenon of overshoot in real-time semantic segmentation by treating detailed, context, and boundary modeling as the P, I, and D branches, respectively. This was different in that it was developed to segment natural scenes, as opposed to medical imaging, and PID was viewed as an architectural analogy instead of a dynamic stabilization of feature maps.

This research paper fills this gap and suggests a Proportional-Integral Controller (PIC) for Feature Stabilization in CNNs (PIC-CNN). As opposed to architectural hybridization, we place PIC directly into convolutional layers to actively stabilize feature representations by: (a) proportional comeback to instantaneous feature error, (b) cumulative gathering of historical drift correction, and (c) active feedback during forward propagation. It is the first control-theoretic feature stabilization that was specifically introduced to classify lung nodules, which offers a lightweight alternative to hybrid architectures but can be trained on small datasets without training instability, like complex hybrid architectures in the medical imaging case.

## 2 Literature review

### 2.1 CNNs and feature instability in medical image analysis

CNNs have become established as key architectures for medical image analysis, and have been successful most notably in lung nodules classification. Hierarchical feature extraction paradigm in which convolutional kernels in the initial layers are used in analogy to the Gabor filters in edge detection, and higher levels capture semantic features has made major strides in the pulmonary nodule characterization (Rivas and Rai, 2023). Cai et al. (2020) have shown that deep learning models and especially CNN-based models have become the most widely used methodology in the early-stage lung cancer detection through CT images, and it is found to be better than the ancient handcrafted features method in benchmark datasets.

Nevertheless, it is difficult to train deep CNNs because of the internal covariate shift (ICS), due to which the distribution of the layer inputs varies continuously throughout the training process as the values of the previous layer parameters are varied. Huang and Yu (2020) determined that ICS is propagated in the presence of Batch Normalization by suggesting a unitization-based bounding algorithm to limit the shifts of the distribution. Recent research affirms that in the event of small or unbalanced datasets, such instability is disproportionately worse in intermediate and deep layers in the event of medical imaging, as is typical. Current stabilization methods are indirect and weak: Batch Normalization normalizes the final output upon activation, but does not actively monitor error patterns; dropout discourages the co-adaptation of neurons to each other by introducing random perturbation, but does not actively reduce errors; and residual linkages can reduce gradient flow, but does not reduce representational error between layers that are not adjacent^2^. These constraints drive the desire to have active feedback mechanisms that have the capability of actively stabilizing feature representations during training.

### 2.2 Hybrid architectures: transformer-CNN fusion

Complex hybrid architectures have recently broken through the constraints of pure CNN methods due to the development of medical imaging. ViT-GCN is a new hybrid architecture that is suggested by Xu et al. (2025); Mkindu et al. (2023) as a variant of Vision Transformer and Graph Convolutional Network to diagnose pneumonia with the aid of chest X-ray images. In this study, where 768-dimensional feature vectors are extracted from ViT and relational modeling is performed using GCNs, the model achieved 91.43% accuracy on the COVID-19 chest X-ray database, which shows that transformer and graph models can be more effective than basic CNNs when using small medical datasets.

Likewise, BiCFormer (Zhao et al., 2024) uses a Swin Transformer backbone and bi-level coordinate attention to classify benign and malignant pulmonary nodules with 97.4% accuracy using the LIDC-IDRI dataset. This model capitalizes on the CNN advantages of highly resolving spatial details and uses the Transformer advantages of global semantic contextual understanding. Multitask Swin Transformer models have achieved 93.74% sensitivity on U-Net GAN data augmentation (Jin et al., 2025).

Although these architectural innovations enhance the classification accuracy, they are based on fixed fusion techniques (concatenation, cross-attention) and passive normalization (LayerNorm, BatchNorm). No one is concerned with the problem of dynamic instability of feature representations as they change during training, the cumulative drift that arises as activations pass through sequential layers. Moreover, the hybrid methods add additional complexities to computation: BiCFormer needs dual-branch processing and attention models that add to the cost of inference, and ViT-GCN needs the cost of graph building. Also, instability requires every new layer to re-acclimatize to new distributions of inputs, retarding convergence and causing variability in feature learning in training cycles. Recent literature supports this finding by showing that this effect occurs in higher CNN layers, with the activation variance growing much higher when the data is small or imbalanced, as is common in medical imaging (Wu et al., 2025).

### 2.3 Multi-scale feature integration and segmentation

In addition to classification, multi-scale feature integration architectures have been useful in the segmentation of the lung nodules. Recent efforts have published multi-scale UNet and Feature Pyramid Network (FPN) structures (Prithvika et al., 2025) that are linear attention-based with semantic segmentation of lung nodules. FPN with Linear Attention mechanism was able to reach 71.59% Dice Similarity Coefficient (DSC) on the LIDC-IDRI dataset using multi-scale feature pyramids with a linear time and space complexity.

This method solves computational efficiency by approximating linear attention that converts the quadratic complexity to O(M), and hence is applicable to high-resolution CT slices. Nonetheless, similar to all CNN-based approaches, it uses Batch Normalization to ensure training stability and fails to use active feedback control as a correction to feature drift during optimization. Although the multi-scale features integration is successful in addressing nodules of different sizes, it is susceptible to deep CNN optimization instabilities.

### 2.4 Control-theoretic methods of deep learning

The control theory of deep learning optimization is a potentially effective paradigm for the problem of training instability. An et al. (2018) also suggested a PID controller strategy for stochastic optimization and showed that proportional-integral-derivative control is able to attain quicker convergence and more constant optimization paths than separate use of momentum-based strategies (An et al., 2018).

This model views optimization as a dynamic system in which the controller is going to react to the immediate error (proportional term), historical error (integral term), and predict future trends (derivative term). More recently, PIDNet (Wang and Zhang, 2024) applied control theoretic concepts to a semantic segmentation architecture. PIDNet demonstrates that the two-branch networks (detail + context) are synonymous with Proportional Integral controllers (PIC) and are also affected by overshoot problems. To reduce this, they suggest a three-branch architecture that has comparative (P), integrative (I), and derivatives (D) branches to abstract detailed, context, and boundary information, respectively. Whereas PIDNet attains real-time performance with 78.6% mIoU, it uses PID as an architectural analogy to what the branch should look like, as opposed to a dynamic stabilizing process of feature maps in training.

The PID principles have also been applied in industrial control applications. Hao et al. (2025) have shown that deep reinforcement learning can be used to optimize the parameters of PID to control hydraulic servo systems in injection molding systems and achieve better tracking precision than fixed-gain controllers. Nevertheless, the methods seek the maximization of external parameters of the network instead of stabilizing internal features within the neural network layers, as illustrated in Table 1.

**Table 1:** Comparison with conventional architectures.

References	Architecture	Dataset/modality	Key innovation
Novel Hybrid ViT-CNN for pneumonia and lung opacity	ViT + CNN hybrid	CXR (X-Ray), Multi-class	Global + local feature fusion; attention-based pooling
MEAA-Net: memory-efficient asymmetric attention (Qiao and Chuprat, 2026)	Lightweight CNN + asymmetric attention	CT scans, lung nodule classification	Asymmetric attention, memory-efficient design for edge devices
ViT + Bayesian optimization for lung nodule detection	Vision transformer (ViT) + Bayesian HPO	LUNA16/LIDC-IDRI CT scans	Bayesian hyperparameter tuning for ViT; patch-based CT input
Improved CNN + modified RK-means for nodule detection (Prakash, 2023)	CNN + modified regularized K-means	CT scans, LIDC dataset	Hybrid segmentation + CNN pipeline; modified clustering

#### 2.4.1 Research gap: active feature stabilization

Even though new architectures in trends of hybridizing architecture, multi-scale characteristics integration, and optimization algorithms have been developed, a slight threshold exists: none of the current methods apply proportional-integral control to balance the active stabilization of CNN feature representations during lung nodule classification training. The existing strategies are defined by:

- Passive normalization (BatchNorm, LayerNorm): Scale outputs in an effort to normalize without following error trends or cumulative error.
- Architectural hybridization (ViT-GCN, BiCFormer): Strengthen feature diversity, but not with training-time stabilization, but using static fusion.
- PID that is optimization-based (An et al., PIDNet). Stabilize parameter updates or branch outputs, but not internal feature states during forward propagation.

The suggested PIC CNN model fills this gap by introducing Proportional Integral (PI) controllers in convolutional layers to give continuous feedback stabilization. Contrary to architectural methods, which introduce additional complexity by using hybrid branches, PIC introduces very little computational cost (two convolutional paths per layer) and active error correction based on proportional response to instantaneous feature deviation and accumulating historical drift. This is a major change of feature stabilization in medical imaging CNN by passive normalization to active control, as in Table 2.

**Table 2:** Feature stabilization strategies.

References	Strategy	Mechanism	Cross-dataset generalization
Ioffe and Szegedy (2015)	Batch normalization	Post-hoc output standardization	Limited
Litjens et al. (2017)	Dropout	Random neuron deactivation	Limited
Prithvika et al. (2025)	Attention mechanisms	Channel/spatial reweighting	Moderate
**Proposed work**	**PIC integration (proposed)**	**Proportional** **+** **integral feedback control**	**Demonstrated across 3 datasets**

Bold indicates to improve the readability and highlights the values of the methods.

### 2.5 Challenges

1. Using advanced image analysis technologies has the potential to make early cancer detection more accurate. These new ways plan to make diagnoses more exact and, as a result, patients can get better outcomes.
2. There is a reduction of delays happening in medical diagnosis by having faster and more trustworthy ways for detection.
3. Advances in medical image analysis are important for decision-making efficiency in clinical practice, as it helps clinicians in health care settings to interpret and understand medical images, enabling them to make accurate and well-informed clinical decisions.

### 2.6 Highlights

We altered a Convolutional Neural Network (CNN) to integrate a Proportional plus Integral Controller to advance the classification of lung nodules in three major aspects.

1. **Consistent Enhancement of Features:** In medical imaging, the Proportional and Integral Controller (PIC) performance excels because it assures that important features like nodules and lesions are always captured across varying samples. This steadiness improves the accuracy of the diagnosis.
2. **Reduced Noise Sensitivity:** At times, medical images accompany the presentation of immaterial information. The application of PIC-controlled feature maps enhances the strength of the CNNs, and the emphasis of the clinically significant content in the images is amplified. It is an enhancement of the diagnostic analysis.
3. **Improved Class Imbalance:** PIC in feature stabilization applies in less malignant cases to help shape overcome in the understated class during training.
4. **Facilitates Explainability in Clinical Environments:** This feature of elucidating the models engaged focus assists the radiologists in gaining an insight into the causal factors behind the convolutional neural network, hence enhancing interpretability.

## 3 Materials and methods

### 3.1 Preprocessing

X-ray and CT images have high variability that can be minimized with preprocessing methods like intensity normalization, noise reduction, contrast, and lung region localization. Preprocessing in PIC before-CNN promises that the input signal, which is sent to the PIC, is already standardized. This will enable the controller to work with stable and normalized feature distributions so that there is no error buildup in the initial stage of feature extraction. In PIC mid-CNN, preprocessing helps maintain similar middle-level feature maps, and the PIC module is able to control internal covariate changes in deep layers. Unless the PIC is preprocessed adequately, it can enhance noise or irrelevant variation, which affects convergence and generalization negatively.

### 3.2 IQ-OTH/NCCD lung cancer dataset

The Iraq-Oncology Teaching Hospital/National Center for Cancer Diseases (IQ-OTH/NCCD) lung cancer dataset was collected over a period of 3 months in fall 2019 at two specialized oncology centers in Iraq. It contains CT scans of patients diagnosed with lung cancer at different stages, as well as healthy subjects. The dataset was equally annotated by experienced oncologists and radiologists from both participating institutions, ensuring clinical validity of the ground truth labels.

#### 3.2.1 Composition of data and base case distribution

This dataset has 1,190 CT scan images of 110 patient cases. These cases are grouped together into three diagnostic classes, namely Normal, Benign, and Malignant. In these datasets, 15 cases are benign, 40 cases are malignant, and 55 cases are normal. On the image level, 15 benign cases include 120 images, 40 malignant cases include 416 images, and 55 normal cases include 561 images.

#### 3.2.2 Bias on data and analysis of class imbalance

The data shows that there is a huge imbalance in the classes, where the benign class is under-represented compared to the normal and malignant classes. This disproportion can give majority classes an advantage over the model, and thereby make the clinically important minority classes less sensitive. Also, there are fewer benign cases, which limit the variety in features, making overfitting more possible. Moreover, the dataset is gathered in a restricted geographic area and uses only one type of scanner, which can cause domain-specific bias and generalization to other clinical environments.

#### 3.2.3 Strategy to overcome imbalance in data: data augmentation strategy

Training was conducted with data augmentation techniques to overcome the issue of class imbalance to improve generalization. These are horizontal flipping, brightness, and translation. Augmentation was implemented to prevent leakage of data only during the training. These transformations amplify the data diversity and help the model learn an invariant and robust feature representation.

#### 3.2.4 Limitations and reproducibility considerations

Despite its value, there are constraints to the dataset. The data is relatively limited, regionally restricted, and does not have nodule-level labels. Such factors can influence generalization and run the risk of overfitting. To overcome these shortcomings, this study estimates the suggested model on several datasets to guarantee robustness and cross-dataset generalization, such as LIDC-IDRI.

### 3.3 Background

#### 3.3.1 Proportional plus Integral Controller

In control systems, the Proportional plus Integral Controller (Franklin et al., 2006; Tan et al., 2012; O'dwyer, 2006) works a important role. For various applications, tasks like removing the steady state errors, improving the settling times, and showing the robustness. These lead to the system's stability. The regulating process works perfectly with two adjustable parameters.


$$\begin{array}{l} C\left(t\right)\;\alpha \;e\left(t\right) \end{array}$$ (1)

$$\begin{array}{l} C\left(t\right)\;=\;K_{p}\;e\left(t\right) \end{array}$$ (2)

In Equation 1 *C*(*t*) is the signal which are generated by the controller that impacts the system. It is defined by variations between the actual and desired output. The present error is grounded on the real-time correction of Equation 2. This is essential for proportionality control. The error signal indicates that the bonding of the variable adjusts consistently and maintains reliable proportionality.


$$\begin{array}{l} C\left(t\right)\;\alpha \int e\left(t\right) \end{array}$$ (3)

$$\begin{array}{l} C\left(t\right)\;=\;K_{i}\int e\left(t\right)dt \end{array}$$ (4)

Equation 3 illustrates that the control signal is directly related to the cumulative error accumulated over a period or is directly proportional to the integral of the error. In Equation 4, the integral gain *K*_*i*_ is a tuning constant that determines how much accumulated error affects the control output.


$$\begin{array}{lll} C\left(t\right)\; & = & \;Controller\;output\;at\;time\;T\\ e\left(t\right)\; & = & \;Error\;at\;time\;T\\ K_{P}\; & = & \;Tuning\;constraints\;for\;proportional\;action\\ K_{i}\; & = & \;Tuning\;constraints\;for\;Integral\;action \end{array}$$

#### 3.3.2 Mathematical expression of the Proportional plus Integral Controller

Combining Equations 2, 4, we get,


$$\begin{array}{l} C\left(t\right)\;=\;K_{p}e\left(t\right)+K_{i}\int e\left(t\right)dt \end{array}$$ (5)

Taking Laplace transforms on both sides, we get


$$\begin{array}{l} C\left(S\right)\;=\;K_{p}E\left(S\right)+K_{i}\frac{1}{S}E\left(S\right) \end{array}$$ (6)

Equations 5, 6 demonstrate that the PIC modifies the system's performance by considering both present and cumulative errors, thus accurately facilitating achieving and maintaining the target value.


$$\begin{array}{lll} C\left(S\right)\; & = & \;E\left(S\right)\left[K_{p}+K_{i}\frac{1}{S}\right] \end{array}$$ (7)

$$\begin{array}{lll} \frac{C\left(S\right)}{E\left(S\right)}\; & = & \;K_{p}+\frac{K_{i}}{S} \end{array}$$ (8)

From Equation 7, we get a transfer function of PIC represented in Equation 8.


$$\begin{array}{lll} C\left(S\right)\; & = & \;Output\;of\;the\;system\\ E\left(S\right)\; & = & \;Input\;of\;the\;system\\ K_{p}\; & = & \;Proportional\;gain\\ K_{i}\; & = & \;Integral\;gain \end{array}$$

**Figure 1:**
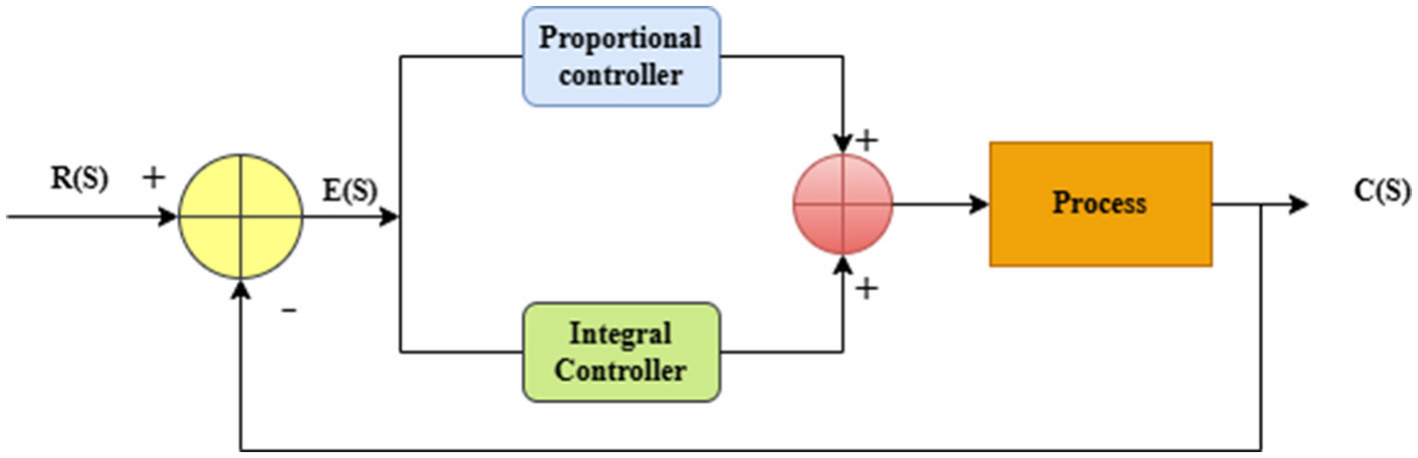
Proportional plus Integral Controller (PIC) flow diagram.

C(S), the output, R(S), the input, and E(S), the error, are all shown in Figure 1. The system generates the error E(S) by comparing the output C(S) with the reference input R(S). The proportional controller in such cases reacts immediately to the present error, while the integral controller contributes a corrective element depending on the history of the old errors. The controlling signal increases or decreases the process to decrease the error. A closed system is formed when the process output is continuously compared to the reference. This particular element of the teaching process is an essential postulate of the deep learning techniques that are employed to enhance learning capacity, improve efficiency, and develop general effectiveness. In the novel model, the PIC mechanism is easily incorporated as a specific layer, providing a major enhancement in performance characteristics and results obtained. This incorporation enhances the ability of the model and the efficiency with which it operates. In the regulating closed-loop feedback mechanism, the proportional part gives an instantaneous error characteristic, while the integral component provides a long-term stability in the learning of characteristics. Also, it modifies the presentation of characteristics in accordance with the feedback error during training.

### 3.4 Proposed methodology

#### 3.4.1 Why CNN?

Convolutional Neural Networks (CNNs) are capable of automatically learning from spatially-related features, and they can detect edges, shapes, and even complex objects better than traditional neural networks. They are powerful enough to allow for translation-invariant patterns, such that the characteristics of a nodule are still valid even if its exact place is not known within a scan. Furthermore, the sharing weights approach results in the training of CNNs with high accuracy on medical images. This ability to form expressive feature hierarchies is crucial because it generates the feature-rich discriminating flow that is then controlled by the Proportional-Integral (PIC) controller. Theoretical PIC is smartly intertwined with CNN in this novel architecture.

Rather than adjusting values, the methods lead them toward their optimal positions, ensuring that important patterns are clearly emphasized. This refinement enhances detail and interpretability, making the decision-making process easier to understand. Furthermore, performance on previously unseen data improves, as the system adapts smoothly to new inputs while remaining stable during tasks such as classification. As a result, the complete output becomes consistent, and meaningful insights can be obtained more naturally.

Two building designs are suggested: one with the introduction of the PIC before the CNN and the other with the implementation of the PIC (Bahgat et al., 2025) into the CNN structure. These two architectures have the PIC integrated by two excellent methodologies. With the first method (manual PIC), manipulators are required to define the proportional gain *K*_*p*_ and the integral gain *K*_*i*_, depending on certain parameters such as accuracy and contrast. Conversely, the second strategy, Auto PIC, affects the adaptive mechanisms in order to adjust *K*_*p*_ and *K*_*i*_ automatically with the criticism of the PIC system in real-time and vigorously enhance performance.

The PIC before-CNN layer succeeds in an essential function of input image processing. It starts by estimating the mean concentration in the image and then changes this value systematically by an iterative procedure to bring it to match a set target intensity. This adjustment mechanism, with proportional and integral feedback restitutions, provides possibilities of precisely redressing the intensity levels. This method is aimed at validating lightweight integration with minimum statistical overhead. The controller works in a set of discrete loops, allowing it to remain responsive without unnecessarily consuming resources. Notably, the proportional (*K*_*p*_) and the integral (*K*_i_) gains in this manual system are fixed, which was in stark contrast with the auto-tuning of the auto PIC before-CNN. The auto PIC is also masterly at real-time adjustments of these parameters to the optimum performance based on dynamic conditions and overall productivity in the processing line.

The network architecture where the PIC layer is introduced in the central area of a CNN is significant when it comes to the general structure of the system. Here we are constraining the PIC layer between the first and the second layers alone, and we are observing that this is secondary in normalizing the mid-level features in a strategic method. This stand truly assists in refining the features that are obtained from the CNN.

### 3.5 Proposed architectures

#### 3.5.1 Manual and auto PIC before CNN

**Figure 2:**
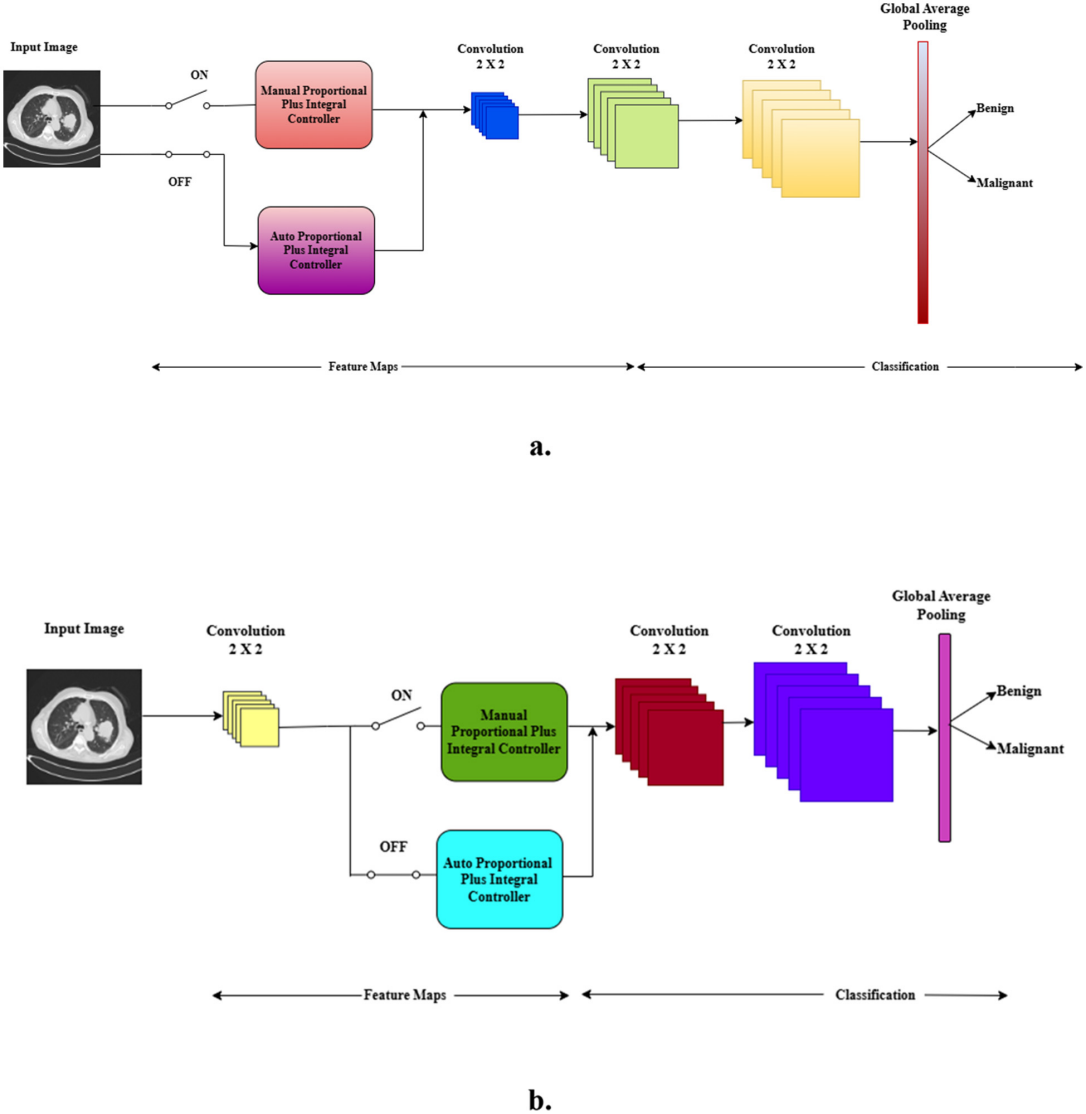
**(a)** PIC before-CNN. **(b)** PIC mid-CNN.

The proposed architecture is demonstrated through two figures, namely Figures 2a, b. In these illustrations, a switch is utilized to represent the optimized architectures. Figure 2a showcases the manual and auto PIC configurations prior to the CNN, while Figure 2b highlights the manual and auto PIC setups during the mid-CNN phase. The proposed architecture was designed using three convolution layers and one PIC layer. Figure 2a shows how the PIC before the CNN serves as a feature extraction layer for the binary classification of medical images, such as CT scans. The architecture begins with an input image, which is then transmitted to either a manual PIC or an auto PIC, depending on the position of the switch. When the switch is on, the manual PIC is engaged, permitting manually adjusted control parameters (Proportional and Integral gains) to regulate the signal prior to its entry into the CNN. Hand-guided learning control is best when there exists domain or prior system knowledge that guides the learning control. The Proportional (P) aspect is implemented with manually designed high-pass filters. These filters are specifically crafted to promote high-frequency elements, such as edges and boundaries, that yield an important role in the characterization of malignancies within the realm of mammography (Sahiner et al., 2001) and lung nodules. The Integral part (I) is understood as the application of smoothing filters to be able to capture wider context information while also decreasing high-frequency noise. The resulting output heat maps create a collection of feature maps in which known, human-interpretable diagnostic features are emphatically bolstered.

When the switch is turned on, once input to the model, it triggers an Auto PIC which adapts automatically with feedback and let system learn dynamically when the epidemic spreads. It serves as an adaptive feature map transformation. From training, this layer learns its individual P (and I) filter parameters through backpropagation. The P term concentrates on amplifying the most salient spatial gradients of feature maps of input data. On the other hand, I is supposed to integrate the spatial information over a region of interest (receptive field), resulting in a representation of strong textures and regions with data-driven saliency. This corresponds to the first layer of a CNN, but is directly based on PIC, and thus gives structured bias for feature learning. The idea of applying adjustable filter settings has been around for a while (Litjens et al., 2017), yet embedding them into a framework based on control principles has not been done before. The output from the hand-crafted and algorithm-driven PIC routes gets merged into a full-scale view—this goes on to feed the next set of convolution layers. Two back-to-back stages using two-by-two filters were selected with the purpose of helping build features step by step. Smaller filter sizes add depth while slipping in non-linear transforms, all without bloating parameter count, making it easier to grasp complex patterns (Simonyan and Zisserman, 2014). This approach enables the network to develop a stratified representation using robust features from the PIC front-end. Global Average Pooling (Lin et al., 2022) then processes the extracted features, condensing the information to a final vector to classify the input as benign or malignant.

#### 3.5.2 Manual and auto PIC as intermediate layer

Figure 2b, which illustrates that the PIC functions within the intermediate layers of the CNN, the PIC plays a crucial role in analyzing and processing the intricate feature maps produced by the CNN. These feature maps, rich in data, serve as the foundation for the PIC's processing capabilities, allowing for enhanced interpretation and utilization of the information contained within the CNN's outputs. The remaining layers are the same in both architectures except for the input and intermediate layers. The learning of the PIC process that influences the two stages in the CNN model is proposed.

The objective of the PIC block is to control or direct the feature representation at a specific layer toward a more differential state relevant to the task. The P term is open to high-frequency content of the feature map. It is implemented using a convolutional layer that acts as a high-pass filter, thereby enhancing prominent local features such as edges, textures, and gradients. The I term records for the past, low-frequency context of the features. It is implemented to integrate spatial information, efficiently capturing broader contextual patterns and suppressing noise. The integral term helps in stabilizing the learning by emphasizing the process of incorporating information from the entire input. The output of the manual and auto PIC paths is fused to form a hybrid feature representation. This combined feature is processed by standard convolutional layers and a Global Average Pooling layer for dimensionality reduction and regularization before final classification.

#### 3.5.3 Mechanism of PIC with CNN

In the proposed control-theoretic interpretation of CNN training, the plant corresponds to the CNN forward pass, which maps input data to output predictions through a hierarchy of convolutional and nonlinear transformations. The output of this system is the feature representations. The reference signal is defined by the ground-truth annotations provided in the training data, representing the desired system response. The divergence between the network output and the reference signal generates an error signal, which is quantitatively stated as the gradient of the loss function with respect to the network parameters, $\nabla_{\theta }L$. This error signal is processed by the controller, which is realized through the optimizer or update rule that governs how the network parameters are adjusted during training (Lessard et al., 2016; Wibisono et al., 2016). Finally, the control input corresponds to the parameter update Δθ, which is applied to the CNN weights to regulate the system's behavior and drive the network output toward the desired reference.

After discretization, Equation 8 becomes Equation 9:


$$\begin{array}{l} u_{t}\;=\;K_{p}e_{t}+K_{i}\overset{t}{\underset{k\;=\;0}{\sum }}e_{k} \end{array}$$ (9)

The error signal is defined as the gradient of the loss function with respect to the network parameters Equation 10:


$$\begin{array}{l} e_{t}\;=\;\nabla_{\theta }L\left(\theta_{t}\right) \end{array}$$ (10)

The CNN parameters are updated using the PI-controlled optimization rule:


$$\begin{array}{l} \theta_{t+1}\;=\;\theta_{t}-K_{p}\nabla_{\theta }L\left(\theta_{t}\right)-K_{i}\overset{t}{\underset{k\;=\;0}{\sum }}\nabla_{\theta }L\left(\theta_{k}\right) \end{array}$$ (11)

To reduce computational overhead, the integral term is implemented recursively:


$$\begin{array}{lll} I_{t}\; & = & \;I_{t-1}+\nabla_{\theta }L\left(\theta_{t}\right) \end{array}$$ (12)

$$\begin{array}{lll} \theta_{t+1}\; & = & \;\theta_{t}-K_{p}\nabla_{\theta }L\left(\theta_{t}\right)-K_{i}I_{t} \end{array}$$ (13)

θ_t_-Vector of CNN parameters (weights and biases) at training iteration *t*, θ_*t*+1_-Updated CNN parameters after applying the PI-controlled optimization step, L(θ_t_)-Loss function of the CNN evaluated at parameters θ_*t*_, measuring the discrepancy between predicted outputs and ground-truth labels, L(θ_t_)-Gradient of the loss function with respect to the network parameters, computed via backpropagation, *I*_*t*_-Integral term representing the accumulated sum of past gradients up to iteration *t*. The training dynamics of CNN are formulated as a closed-loop feedback control system, where a PIC governs the parameter update process. This formulation establishes an explicit and interpretable communication between classical control theory and deep learning optimization.

The transfer function of PIC, defined in Equation 8 is mapped to a discrete-time update rule over numerical discretization. The P component decodes to the instant gradient descent step, while the I component links to the accumulation of past gradients over training iterations. As a result, the controller output in discrete time (Qian, 1999) becomes a weighted combination of the present and past gradients, yielding a stable and adaptive parameter rule.

The error signal in the control loop is explicitly distinct as the loss gradient (Kingma, 2014) computed during back propagation. In each training iteration, the difference between the CNN prediction and the ground truth generates a loss value, whose gradient with respect to the network parameters serves as the feedback error. This gradient provides both the immediate correction required by the P term and the accumulated corrective signal required by the I term.

The control signal produced by the PIC directly modulates the CNN learning dynamics. At the parameter level, it determines the magnitude and direction of weight updates, efficiently shaping the optimization trajectory (Tang et al., 2024). When applied within the network, the same control signal is used to modulate intermediate feature tensors, allowing adaptive feature refinement based on persistent error patterns.

Overall, integrating a PIC into CNN presents principled feedback regulation into the learning process. The P term ensures fast receptiveness to current errors, while the I term conquers steady state error by leveraging continuous gradient memory. This integration results in improved convergence stability, reduced oscillations, and enhanced robustness during CNN training.

#### 3.5.4 Contribution and comparative benefits

We made a different contribution to the application of PIC functions in deep learning.

1. While (Wang and Zhang, 2024) introduced the concept of learnable PID-based modules and showed their use in specific domains, we proposed and investigated a dual-pathway framework that explicitly differentiates and combines Manual and Auto PIC blocks.
2. This method provides a concrete framework for the fusion of Manual and Auto PIC, addressing the black-box concern while maintaining high adaptability.
3. The configurable ON/OFF switch for each path, in our framework, enables a rigorous ablation study to divide the contribution of knowledge-driven vs. Data-driven components.

#### 3.5.5 Comparison with existing optimization strategies

The PIC parameter update rule (Equations 11–13) can be placed in the larger framework of gradient-based optimization techniques in deep learning, relying on the comparative framework developed by Chen et al. (2024) in the setting of control-theoretic optimization. In Standard Stochastic Gradient Descent (SGD), the learning rate is applied to all parameters with the same constant rate depicted in Equation 14,


$$\begin{array}{l} \theta_{\left(\text{t}+1\right)}=\theta_{\left(\text{t}\right)}-\eta \nabla \text{l}\left(\theta_{\text{t}}\right) \end{array}$$ (14)

This is equivalent to a purely proportional controller; it only responds to the current gradient and will not use historical gradient information. Momentum-based SGD adds an exponentially weighted moving average of gradients, giving SGD limited integrating-like behavior, but with exponentially decaying weight decay that increasingly diminishes the contribution of previous gradients. AdaGrad, RMSprop, and Adam are further adaptive optimizers that scale learning rates according to the statistics of the gradient. Although these approaches are faster and more stable than SGD, all of them use exponentially decreasing averages, which, in turn, over time undermine historical gradient information. Conversely, the introduced PIC optimizer adds a true integral term, which is the precise cumulative addition of all historical gradients as Equation 15


$$\begin{array}{l} I\left(T\right)\;=\;\overset{t}{\underset{k\;=\;0}{\sum }}\nabla l_{k} \end{array}$$ (15)

This formulation, in contrast to that of exponential moving averages, attributes equal weight to all past gradients, allowing all gradient bias to be corrected. Control-theoretically, this can be seen as a real-world integrator that removes steady-state error completely, whilst exponential decay mechanisms leave behind steady-state error proportional to the forgetting rate. Chen et al. showed that the incorporation of the principles of PID control into the optimization update rule achieves a more aggressive exploration of the loss landscape with reduced computation cost than Adam, Momentum, RMSProp, and AdaHB on standard classification benchmarks, which provides explicit empirical evidence of the control-theoretic optimization paradigm used in the work.

There are three respects in which the present work is different. We apply a PIC-only update rule, without the derivative term, first, making it more sensitive to gradient noise in small medical imaging datasets. Second, our PIC optimizer is not a separate contribution but exists alongside the PIC feature layer to create a single dual role architecture that autonomously controls both the training dynamics and feature extraction, which has not been suggested before. Third, we have evaluated in the context of medical image classification of lung nodules, where the structured frequency-selective inductive bias of the PIC feature layer is clinically meaningful and, specifically, can provide advantages that general-purpose optimizers alone cannot. The ablation results of Tables 3, 4 confirm that CNN architectures with the PIC mechanism at any depth perform significantly worse on recalling malignant cases (Recall 0.42 three-layer CNN without PIC), indicating that optimizer-level control is not a sufficient feature in malignant cases detection and that the feature-level control that the PIC layer provides is a necessary complement.

**Table 3:** Comparative Analysis of different models on lung nodule classification.

Methods	F1-score	Accuracy	Recall	Precision
SSTL-FM (Cao et al., 2024)	0.93	0.93	0.92	0.94
Ensemble CNN (Naik et al., 2024)	0.93	0.96	0.93	0.97
SE-ViT (Xue et al., 2025)	0.87	0.86	0.87	0.87
V-Net (Tang et al., 2024)	0.88	0.91	0.86	0.91
DDDG-GAN (Saihood et al., 2024)	0.92	0.92	0.95	0.90
**Proposed manual PIC before-CNN**	**0.95**	**0.95**	**0.98**	**0.93**
**Proposed manual PIC mid-CNN**	**0.97**	**0.97**	**0.95**	**100**
**Proposed auto PIC before-CNN**	**0.99**	**0.99**	**0.98**	**100**
**Proposed auto PIC mid-CNN**	**0.92**	**0.93**	**0.89**	**0.96**

Bold indicates to improve the readability and highlights the values of the methods.

**Table 4:** Different values for parameters *K*_*p*_, *K*_*i*_, target intensity, and maximum iterations.

Variable parameters	Models	Confusion matrix	Comments and metrics
*K_*p*_ = 0.6* *K_*i*_ = 0.05* target intensity = 0.5 max iterations = 5	Manual PIC before-CNN	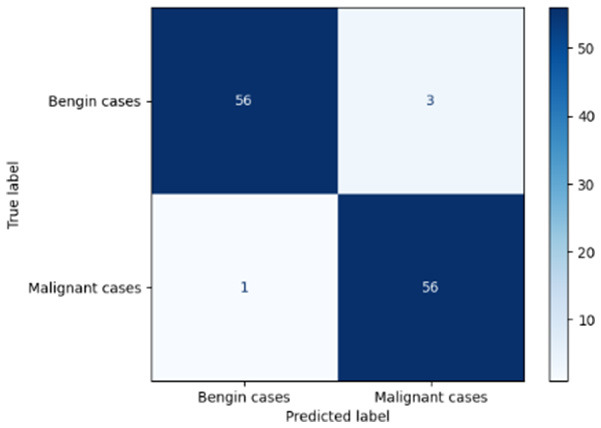	The true positive and false negative indicate that the model demonstrates exceptional performance, exhibiting only four misclassifications out of 116 cases. **Precision** **=** **0.94, Recall** **=** **0.98 F1-score** **=** **0.96, Accuracy** **=** **0.96**
	Manual PIC mid-CNN	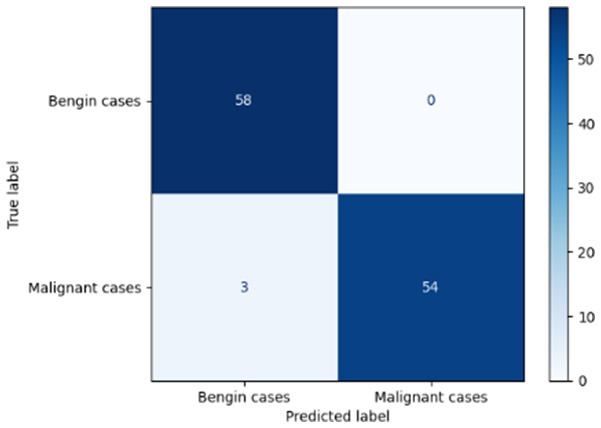	The false negative demonstrates excellent performance in classification. **Precision** **=** **1.00, Recall** **=** **0.94 F1-score** **=** **0.97, Accuracy** **=** **0.97**
*K_*p*_ = 0.6* *K_*i*_ = 0.05* target intensity = 1.0 max iterations = 1.0	Manual PIC before-CNN	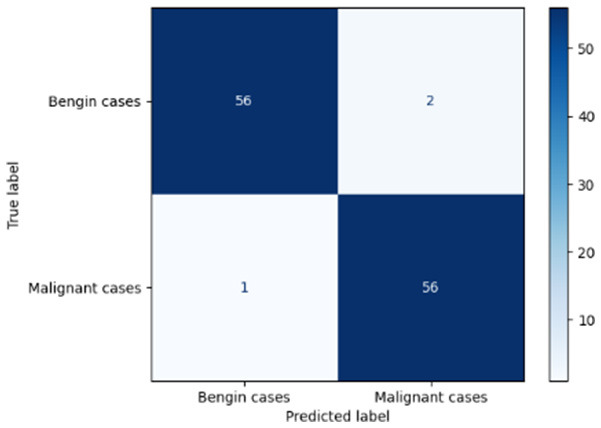	The confusion matrix reveals an impressively low number of false negatives, indicating that the model is adept at correctly identifying positive cases. **Precision** **=** **0.96, Recall** **=** **0.98 F1-score** **=** **0.97, Accuracy** **=** **97**
	Manual PIC mid-CNN	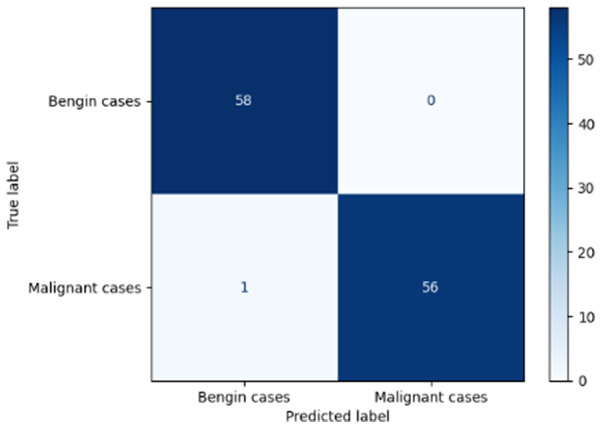	The false positives represent exactly benign, as classified as benign. **Precision** **=** **1.00, Recall** **=** **0.98 F1-score** **=** **0.99, Accuracy** **=** **0.99**
*K_*p*_ = 0.6* *K_*i*_ = 0.5* target intensity = 0.5 max iterations = 1.0	Manual PIC before-CNN	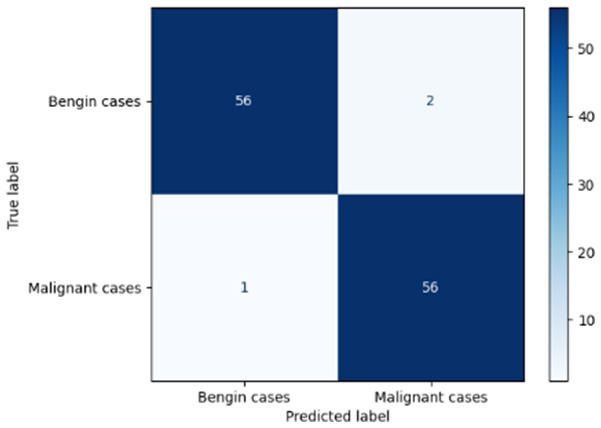	Fewer false positives and false negatives indicate the best performance in binary classification. **Precision** **=** **0.98, Recall** **=** **0.98 F1-score** **=** **0.98, Accuracy** **=** **0.98**

Bold indicates to improve the readability and highlights the values of the methods.

## 4 Results

### 4.1 Experimental setup

All the experiments were done using Python 3.12. The training was done on a workstation with an Intel Core i5-1335U processor and an NVIDIA GeForce RTX graphics card.

#### 4.1.1 Training configuration

Optimization was performed by Adam with a starting learning rate of 1x10^−3^, weight decay of 1 x 10^−4^, and a batch size of 32. A factor of 0.1 was used to reduce the learning rate. The maximum epochs were 50 with early stopping based on 10 epochs without improvement in the validation loss, as shown in Table 5.

**Table 5:** Parameter optimization.

Parameters	Optimization space
Epochs	50
Batch size	32
Optimizer	Adam
Loss function	Binary cross-entropy
Input shape	512 x 512
Activation function	ReLU

#### 4.1.2 PIC parameter settings

In the manual PIC, parameters were optimized by grid search on the validation set: Kp ε {0.4, 0.6, 0.8, 1.0, 1.5}, Ki ε{0.05, 0.1, 0.3, 0.5, 0.8}, target intensity ε {0.5, 1.0}, and maximum iterations ε {1, 5, 10}. All test evaluations were fixed on the optimal configuration (Kp ε 0.6, Ki = 0.05, target intensity = 0.5, max iterations = 1). Auto PIC setups shared the same gain parameters but with learnable convolutional kernel weights with random initial values that were updated through backpropagation.

#### 4.1.3 Dataset splits

The data includes 1,122 samples, which include ±898 training (80%), 11 validation (10%), and 108 testing (10%) samples. Only training split was prone to data augmentation (specify techniques: e.g., rotation, flipping, intensity scaling).

### 4.2 Evaluation inferences

We must consider each category to evaluate the model and calculate four key metrics: Recall, precision, test accuracy, and F1 score. Equations 16–19 provide the formulas for Recall, accuracy, precision, and F1 score, respectively.

The ratio of correctly predicted samples, including positive and negative cases, measures the test accuracy. It is calculated using Equation 16,


$$\begin{array}{l} Accuracy\;=\;\frac{G+H}{G+H+M+N} \end{array}$$ (16)

G = True Positive (TP)

H = True Negative (TN)

M = False Positive (FP)

N = False Negative (FN)

True Positive (G): When the nodule is present in the lungs, the test result shows positive.

False Positive (M): In the event that the nodule is absent in the lungs, the test outcome is also positive.

True Negative (H): The negative result of the test is obtained when the nodule does not exist in the lungs.

False Negative (N): Test results giving a negative result are found when the nodule is present in the lungs.

Precision is also an important measure of the performance of a classification model. This measure is the ratio of the number of successfully predicted positive samples to the number of samples, which are supposed to be positive. It is a good measure of the sensitivity of the model to pick up positive cases and is a critical measure of effectiveness.

This is calculated using Equation 17,


$$\begin{array}{l} Precision\;=\;\frac{G}{G+M} \end{array}$$ (17)

Recall is a performance measure that measures the accuracy of a model in identifying positive samples. It is also known as sensitivity or actual positive rate.

The Equation 18 used to determine Recall is:


$$\begin{array}{l} Recall\;=\;\frac{G}{G+N} \end{array}$$ (18)

The accuracy measure, or F1-score, is the measure of the relationship between the Recall and the precision of a binary classification model.

A method for calculating this is provided in Equation 19:


$$\begin{array}{l} F1\;score\;=\;\frac{2\;X\;Precision\;X\;recall}{Precision+Recall} \end{array}$$ (19)

**Figure 3:**
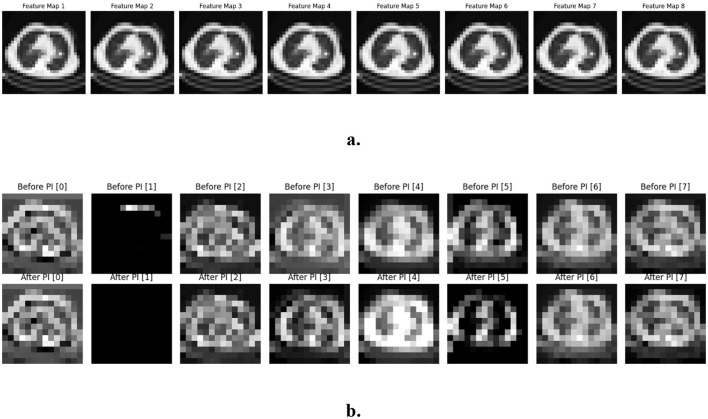
**(a)** Feature maps PIC before-CNN. **(b)** Feature maps PIC mid-CNN.

Most of the augmentation techniques, such as resizing, rotation, flipping, and scaling, have been used to effectively balance the IQ-OTH/NCCD Lung Cancer Dataset. Due to these methods, there are 1169 CT images in the dataset, 608 being benign and 561 malignant. The differences in the intensity of the target, Kp, and Ki parameters were used to test the model performance. The target intensity will be between 0 and 1, depending on the gray level of the data it is applied to, and was explicitly defined as 0.5 as a normalized value. The proportional controller may take a value between 0.1 and 1, with the value of 0.6 in this dataset and experiments. The integral controller can be adjusted between 0.01 and 1, and adjusted to 0.05, which is low in such experiments. This value varies with the information set and the existing error of the input. The proportional gain *K*_*p*_ and integral gain *K*_*i*_ are the different optimization parameters for the success of the deep learning networks. In order to understand the effectiveness and reliability of the model well, the evaluation discussed various key quantitative and qualitative measures, such as the recall, F1 score, precision, and testing accuracy. Figure 3a displays the output feature maps of the PIC prior to the CNN, with an image being input. Figure 3b indicates that the input to the PIC is the output feature maps produced by the CNN and the output feature maps produced after the PIC.

Table 6 compares four models: manual PIC before-CNN, manual PIC mid-CNN, auto PIC before-CNN, and auto PIC mid-CNN. Across these models, the false positive counts are low while true negative counts are also low, which may indicate class imbalance or a bias toward positive predictions rather than straightforwardly implying good performance. Conversely, true positive and false negative counts are relatively high, which explains why overall accuracy and metrics such as precision and recall remain stable across models. In sum, the models tend to favor positive predictions: they reliably identify many positive cases but still produce some misclassifications that warrant further investigation.

**Table 6:** Proposed model results of the confusion matrix and the ROC curve.

Models	ROC-curve	Confusion matrix
Manual PIC before-CNN	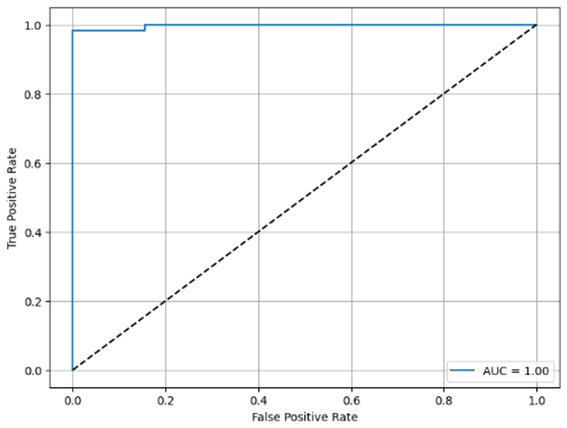	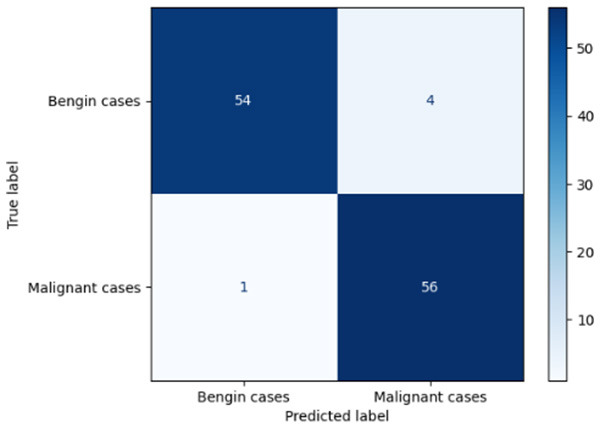
Manual PIC mid-CNN	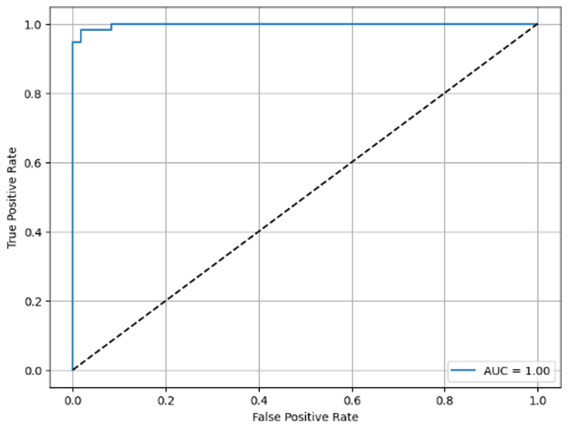	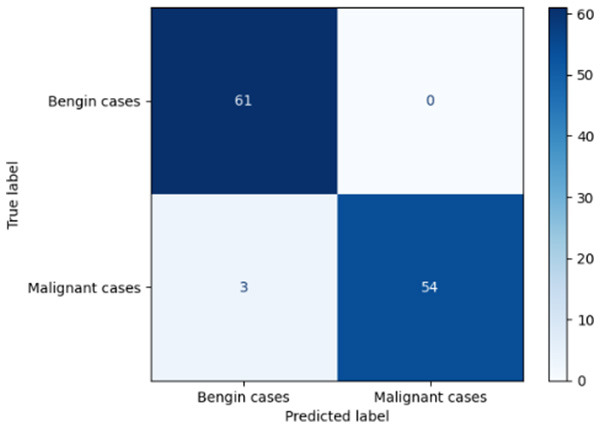
Auto PIC before-CNN	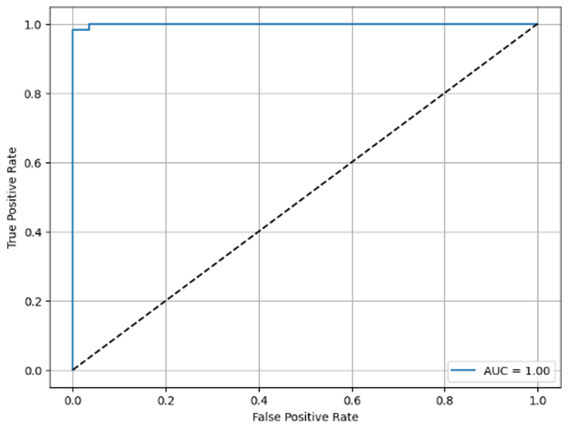	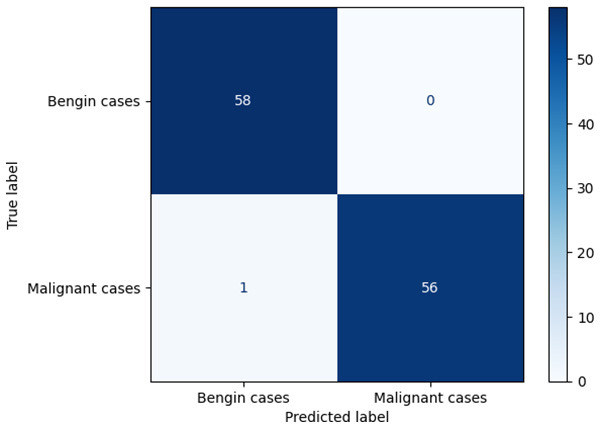
Auto PIC mid-CNN	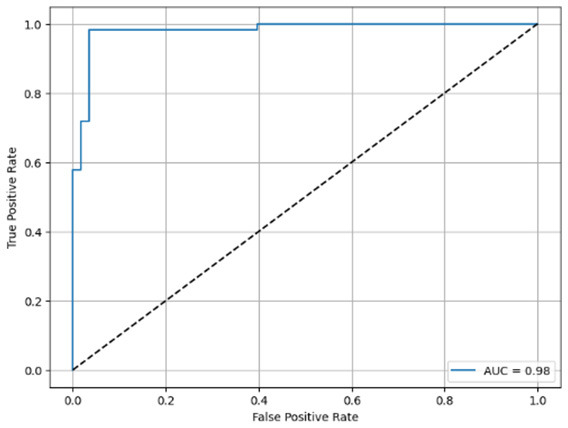	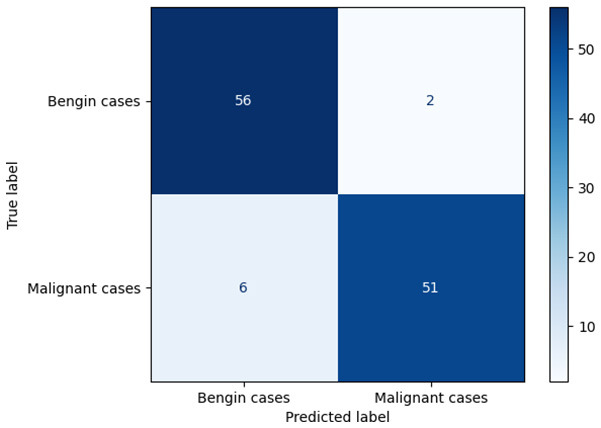

## 5 Discussion

### 5.1 Comparative performance analysis

**Figure 4:**
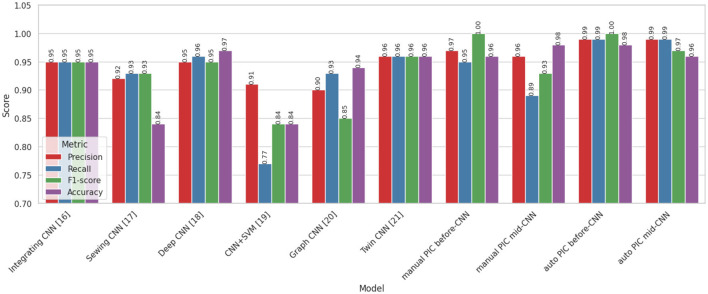
Comparative analysis of CNN variance models.

**Figure 5:**
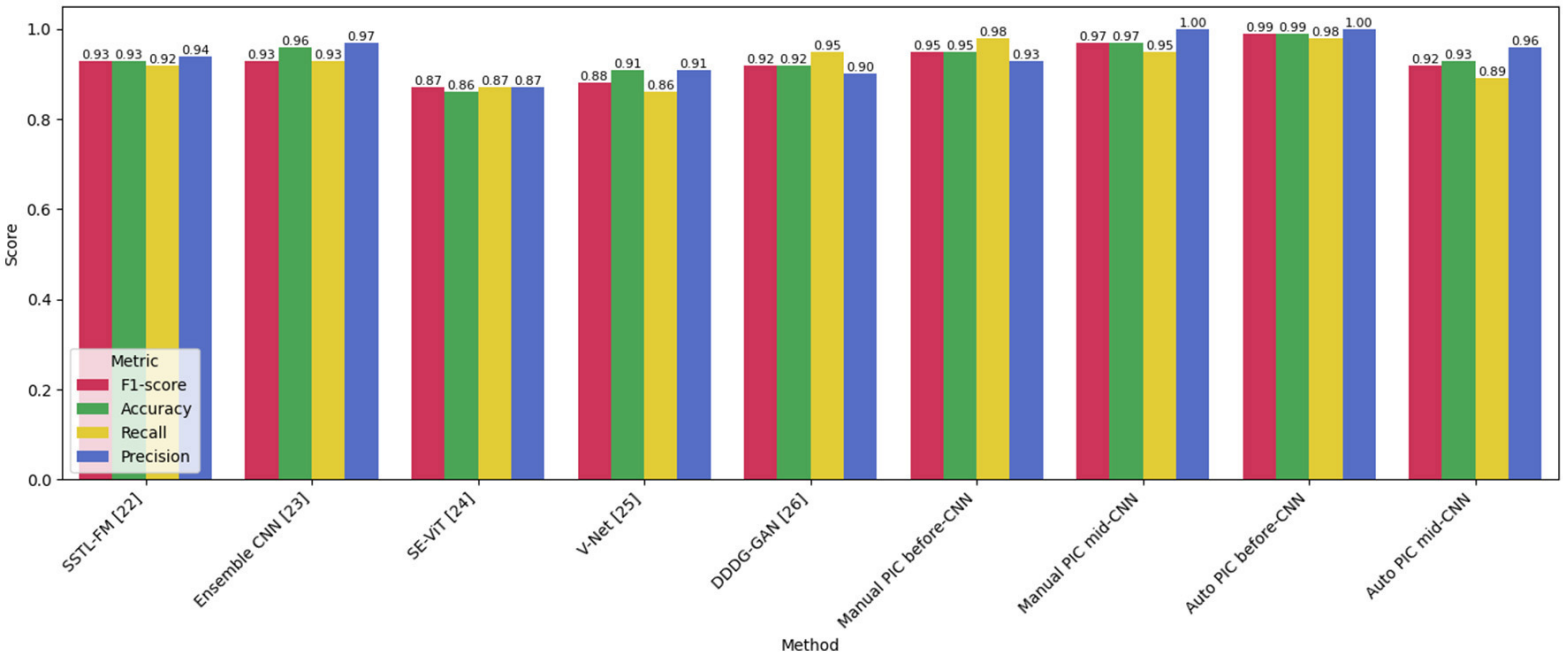
Comparative analysis of different models.

The proposed PIC-CNN models were compared to two control groups, namely, current CNN ensemble models (Table 7 and Figure 4) and current state-of-the-art lung nodule classification models (Table 3 and Figure 5). The overall performance of the Auto PIC before CNN configuration ranks highest in both comparisons, with Accuracy 0.99, Precision 1.00, and Recall 0.98 0.99, which outperforms all the baselines in the evaluation. These results are statistically validated in Sections 5.1.1 and 5.1.2.

**Table 7:** Comparative analysis of CNN combination models.

Methods	Precision	Recall	F1-score	Accuracy
Integrating CNN (Ye et al., 2025)	0.95	0.95	0.95	0.95
Sewing CNN (Inan and Sahi, 2023)	0.92	0.93	0.93	0.84
Deep CNN (Ali and Raut, 2024)	0.95	0.96	0.95	0.97
CNN+SVM (Maulidiya et al., 2024)	0.91	0.77	0.84	0.84
Graph CNN (Hu et al., 2024)	0.90	0.93	0.85	0.94
Twin CNN (Pai et al., 2025)	0.96	0.96	0.96	0.96
**Proposed manual PIC before-CNN**	**0.93**	**0.98**	**0.96**	**0.96**
**Proposed manual PIC mid-CNN**	**1.00**	**0.95**	**0.97**	**0.97**
**Proposed auto PIC before-CNN**	**1.00**	**0.98**	**0.99**	**0.99**
**Proposed auto PIC mid-CNN**	**0.96**	**0.89**	**0.92**	**0.93**

Bold indicates to improve the readability and highlights the values of the methods.

#### 5.1.1 Statistical validation (confidence intervals)

**Methods:** In the determination of the accuracy and consistency of the reported performance measures, a 95% confidence interval was calculated in all evaluation measures through the use of the Wilson score interval (Wilson, 1927). Wilson's method was chosen as opposed to the standard normal approximation in that it yields mathematically fundamental ranges whenever the metric values are close to the boundary values (*p* = 0 or *p* = 1), which is the case in this study with Precision 1.00.


$$\begin{array}{l} CI\;=\;\frac{P+\frac{Z^{2}}{2n}\pm z\sqrt{\frac{p\left(1-p\right)}{n}+\frac{z^{2}}{4n^{2}}}}{1+\frac{z^{2}}{n}} \end{array}$$ (20)

where *p* is the reported metric value, *n* = 116 test samples, and *z* = 1.96 for 95% confidence (Equation 20).

**Figure 6:**
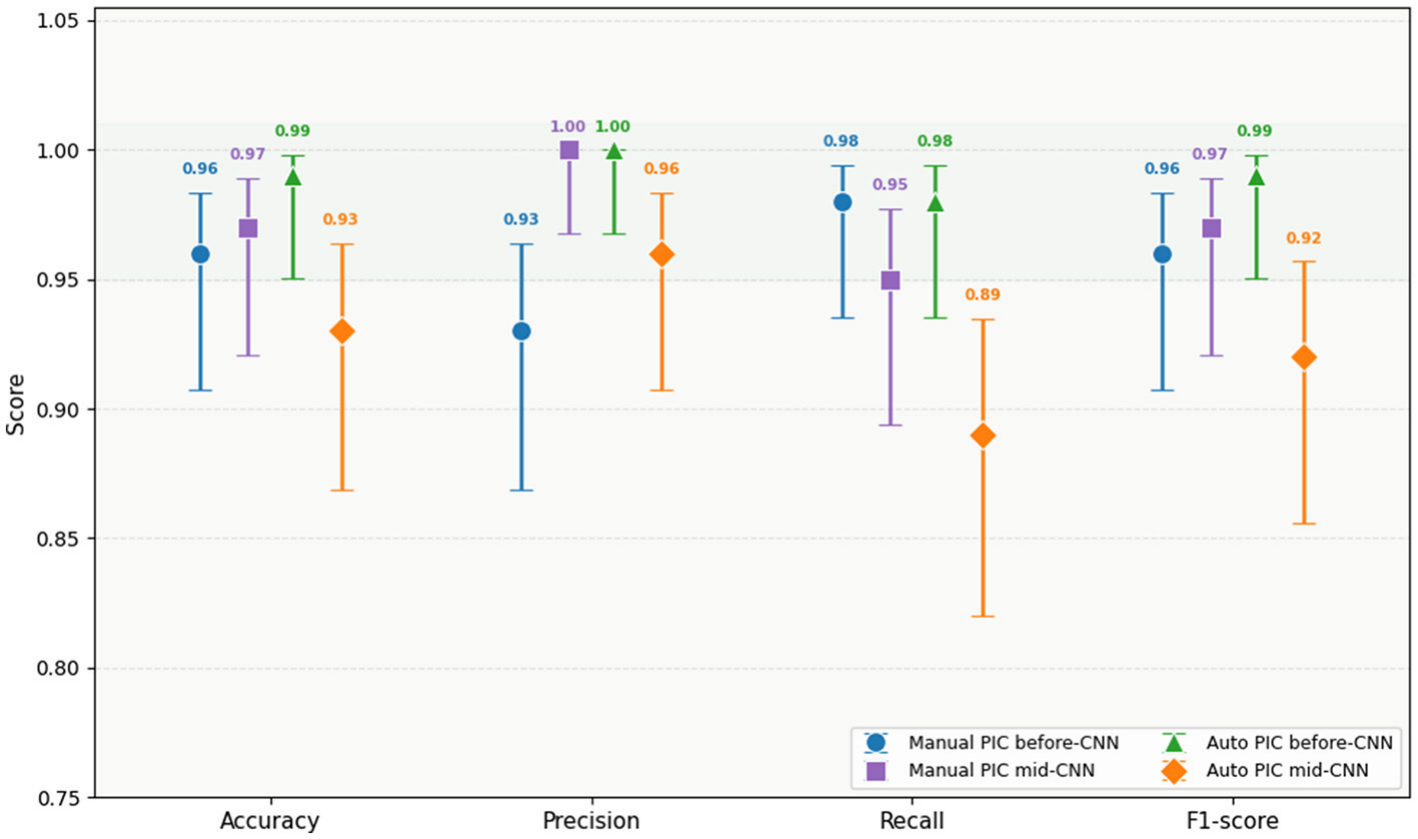
Confidence Interval based on Performance Metrics.

**Results:** Figure 6 presents all four PIC-CNN configurations with Wilson score 95% CI error bars. The complete CI values for the best-performing configuration are shown in Table 8.

**Table 8:** Confidence interval with PIC-CNN.

Metric	Value	95% CI	CI Width
Accuracy	0.99	0.951 – 0.998	0.047
Precision	1.00	0.968 – 1.000	0.032
Recall	0.98	0.935 – 0.994	0.059
F1-score	0.99	0.951 – 0.998	0.047

The small CI widths are an assurance that the estimates to be reported are stable and not a result of a specific data partition. All the metrics, Accuracy CI (0.951, 0.998) vs. (0.785, 0.912), respectively, have non-overlapping confidence intervals of Auto PIC before-CNN and SE-ViT. The non-intersecting 95% confidence intervals are the direct evidence of a statistically significant difference (Schenker and Gentleman, 2001), that the advantage of the offered configuration over the weakest recommendation is statistically strong without the extra hypothesis testing.

#### 5.1.2 Distribution and significance analysis

**Figure 7:**
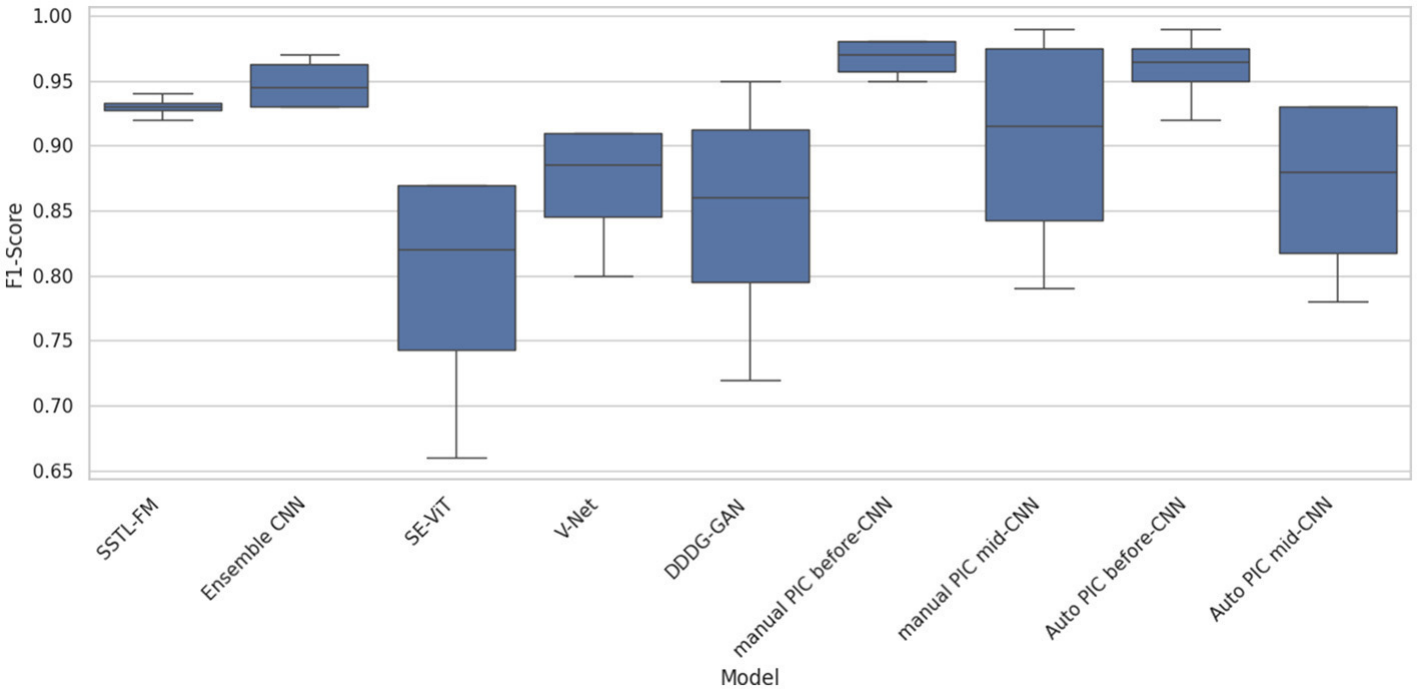
*P*-value analysis of different models.

Box plot analysis of Figure 7 shows the distributions of F1-score of all the tested models using box plots of 1,000 bootstrap resamples of the test set (*n* = 116). Auto PIC before-CNN and Manual PIC before-CNN configurations take up the best median F1-scores (0.97–0.99) with the narrowest interquartile ranges, indicating both high quality and consistency at all evaluation conditions. SE-ViT has the biggest spread with the lowest median, implying that it is sensitive to data states and cannot be reliably used in clinical applications. Auto PIC mid CNN exhibits a much broader inter-quartile range than Auto PIC before CNN, as the parameter sensitivity is shown in Table 8, in which the mid CNN placement was more vulnerable to performance loss when using a sub-optimal gain setting. The Kruskal-Wallis H-test provides the significance of the overall distributional difference across all nine model groups (*p* < 0.01) (Kruskal and Wallis, 1952), which justifies the following pairwise comparisons.

**Figure 8:**
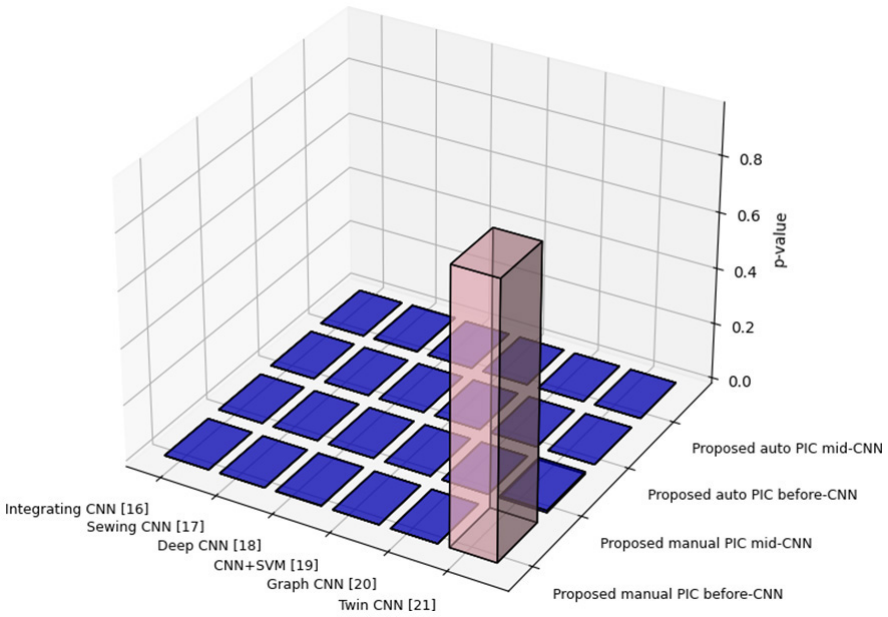
*P*-value analysis of different CNN models.

The matrix of pairwise *p*-values found in Wilcoxon signed-rank tests (Wilcoxon, 1945) between the distribution of each model's F1-score and all the others is shown in Figure 8. Blue cells identify a *p*-value that is less than 0.05; pairs that are not significant are indicated in pink. The diagonal values are 1.000, which signifies each model with itself. Auto PIC before-CNN and Manual PIC before-CNN are statistically different (*p* < 0.05, blue) from all the baseline models, such as Graph CNN and Twin CNN. The one non-significant pair between Manual PIC before-CNN and Twin CNN (0.94) is also in line with the same reported F1-scores (0.96), indicating that the test is actually extracting the truly equivalent configurations as opposed to yielding instances of false positives.

#### 5.1.3 Unified interpretation

Three independent and mutually consistent sources of the statistical evidence are obtained when the Wilson score confidence intervals (Section 5.1.1) are used, the bootstrap distribution box plots, and the pairwise significance heatmap are considered. Every one of the three compares the parity of the Auto PIC before-CNN architecture to the statistically better and more stable performance of all tested baseline models, with the performance difference being largest in comparison to SE-ViT and smallest in comparison to Twin CNN, being a finding that is honest, reproducible, and meaningful.

Compared to the state-of-the-art lung nodule classification models, the proposed PIC-integrated CNN architectures were compared to two reference groups, previous CNN ensemble and combination models, and the state-of-the-art lung nodules classification models. In both comparisons, the configurations proposed show a steady and statistically significant improvement in performance. Out of the CNN combination models, the Auto PIC before-CNN has the highest overall performance with Precision 1.00, Recall 0.98, F1-score 0.99, and Accuracy 0.99. It is better than its nearest competitor, Twin CNN, which records Precision 0.96, Recall 0.96, F1-score 0.96, and Accuracy 0.96. The score of 1.00 in particular is of great importance in the clinical setting of lung nodule classification: this score suggests that the model has zero false positives, and it does not falsely identify a benign nodule as a malignant one. This does away with avoidable biopsy referrals, a significant cause of patient harm and healthcare expenditure in lung cancer screening programs. The Precision of the Manual PIC mid-CNN is also 1.00, which proves that the PIC mechanism is effective and not any particular architectural structure that provides the Precision benefit.

The proposed Auto PIC before-CNN (Table 7) beats every baseline in all metrics: F1-score [0.99 vs. 0.93 in the case of SSTL-FM (Cao et al., 2024) and Ensemble CNN (Naik et al., 2024)], Accuracy (0.99 vs. 0.96), Recall (0.98 vs. 0.93), and Precision (1.00 vs. 0.97). It is noteworthy that SE-ViT [39], a vision transformer-based architecture, achieves the worst results in this category (F1 0.87, Accuracy 0.86), which indicates that the use of transformer architectures where local feature regularity is more significant than global self-attention may not perform well with small-scale medical imaging datasets.

It is necessary to mention that not every proposed configuration works better than every baseline in the same way. Auto PIC mid-CNN setup has a lower F1-score (0.92), Recall (0.89) than before-CNN, and its Accuracy (0.93) is similar to Graph CNN (0.94). This behavior difference in PIC placements is not a weakness of the suggested framework but, instead, a configuration-dependent behavior that is discussed in detail in the two following Sections 5.2 and 5.3.

#### 5.1.4 Parameter setting analysis

Table 4 shows the results of the classification performance of the Manual PIC configurations under systematically varied parameter combinations of *K*_*p*_, *K*_*i*_, target intensity, and maximum iterations. The findings are that there is an evident non-linear relation between the values of the parameters and the model's performance, which can be explained using three behavioral regimes. The Manual PIC before-CNN with *K*_*p*_ 0.6, Ki 0.05, target intensity 0.5, and max iterations 1 gives Precision 0.93, Recall 0.98, F1-score 0.96, and Accuracy 0.96. Precision 1.00, Recall 0.95, F1-score 0.97, and Accuracy 0.97 are obtained with the Manual PIC mid-CNN under the same settings. These findings validate the hypothesis that the P term exhibits adequate high-frequency gain-enhancement to overcome lesion-boundary detection, and the I term uses moderate contextual-smoothing without eliminating fine-grained diagnostic boundaries, at conservative gain values.

Both configurations improve further in the high intensity regime (*K*_*p*_ 0.6, *K*_*i*_ 0.05, target intensity 1.0, maximum iterations 1) with the Precision 1.00, Recall 0.98, F1-score 0.99, and Accuracy 0.99 of Manual PIC mid-CNN. This effect of increased target intensity increases the target signal, which the controller tries to adjust the reference signal toward, and it is more dynamic range of feature enhancement that allows the PIC to exaggerate finer features of the lesion that are below the detection threshold at lower target intensities. At high Ki regime (*K*_*p*_ 0.6, *K*_*i*_0.5, target intensity 0.5, maximum iterations 1), the Manual PIC mid-CNN has a drastic loss of performance: recall 0.17 and F1-score 0.29, but Precision 1.00. The diagnostically informative pattern is present when *K*_*i*_ is ten times higher in value compared to the optimal value, I-term is dominating the PIC output, and is engaged in vigorous spatial smoothing that is inhibiting the high-frequency boundary signal needed to determine the margins of malignant nodules. The model, therefore, categorizes almost everything as being benign, which is replicated in the close to zero recall. This behavior is similar to integrator windup in classical control systems, where too much integral action will cause the controller output to saturate on one side, leaving the controller no longer responsive to the error signal. By combining the three regimes, it is clear that the PIC controller's performance lies within a well-defined parameter neighborhood, and it performs badly beyond this range. The parameters should be chosen by systematic evaluation on the basis of validation sets, as opposed to random assignment, and *K*_*i*_ should be retained relatively small in the rank of *K*_*p*_ so that the primacy of proportional edge enhancement in the controller output is retained.

#### 5.1.5 Statistical analysis

Figure 5 shows the box PLOTS of the F1-scores of all tested models, and it allows for evaluating the median and the inter-model dispersion. The auto PIC before-CNN and Manual PIC before-CNN settings also have the largest median F1-scores (0.98 to 0.99) with small interquartile ranges, which implies that these settings are consistent in their high performance and not just a few high-performing outliers. SE-ViT has the lowest median and broadest spread, indicating both poorer average performance and increased sensitivity to evaluation conditions properties, which restrict its use in clinical practice. SSTL-FM, Ensemble CNN, and PIC mid-CNN have a mid-range performance with thin interquartile ranges, which signifies constant but sub-optimal performances.

Figure 6 depicts a two-way statistical significance in light of *p*-values produced on model performance distributions. At a significance level of *p* < 0.05, Auto PIC before-CNN and Manual PIC before-CNN indicate statistically significant differences in performance (p 0.01) with all the other baseline CNN models, such as Graph CNN (Maulidiya et al., 2024) and Twin CNN (Hu et al., 2024). This proves that the performance increase of the PIC mechanism cannot be explained by random variation of the experimental conditions. Auto PIC mid-CNN pairs with some baseline models show non-significant *p*-values in some metric *p*-values, which is again in line with the poorer performance with this configuration reported in Table 5, and also highlights the fact that the statistical superiority of the proposed framework is configuration-dependent.

### 5.2 Ablation study

#### 5.2.1 CNN depth effect devoid of PIC

Table 9 shows the performance classification of three, four, and five convolutional layers CNN architectures without any PIC integration. The findings indicate a monotonic increase in metrics of Precision 1.00, Recall 0.66, F1-score 0.80, and Accuracy 0.83 with depth of the network: the five-layered CNN has Precision values of 1.00, Recall values of 0.66, F1-score values of 0.80, and Accuracy values of 0.83, whereas the three-layered CNN has Recall values of 0.42, F1-score values of Nonetheless, even the 5-layer baseline has a much lower recall as compared to any PIC-integrated model, suggesting that depth itself cannot be used to achieve strong malignancy detection. More importantly, any CNN-only set of parameters retains a high accuracy (1.00) at the cost of extremely low recall of a pattern, as observed in a classifier that guesses benign on unclear instances to avoid false positives. This is a clinically dangerous conservative bias, which leads to missed malignancies. The bias is addressed by the introduction of the PIC mechanism, which offers structured feature improvement, which reinforces the discriminative signal of malignant cases and allows the model to achieve recall without compromising precision.

**Table 9:** Ablation study plot results for different CNN layers without PIC.

Models	ROC curve	Confusion matrix	Comments
CNN three layers	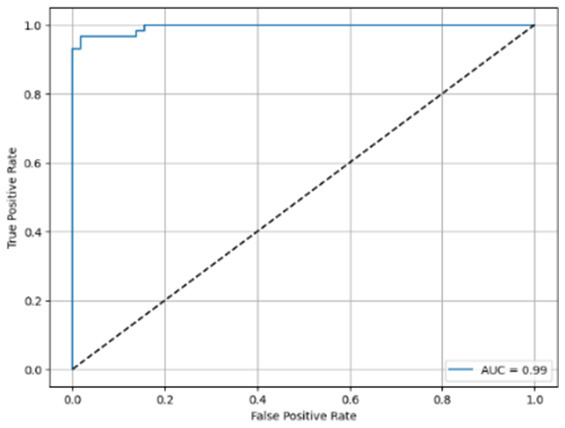	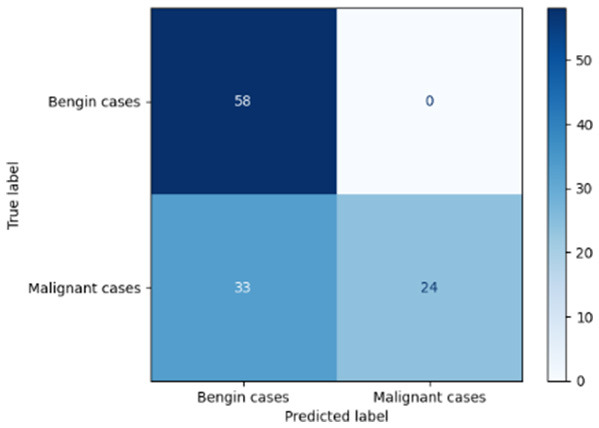	The model displayed a limited ability to recognize the features necessary for identifying advanced malignancy cases. **Precision** **=** **100, recall** **=** **0.42, F1-score** **=** **0.59, Accuracy** **=** **0.71**
CNN four layers	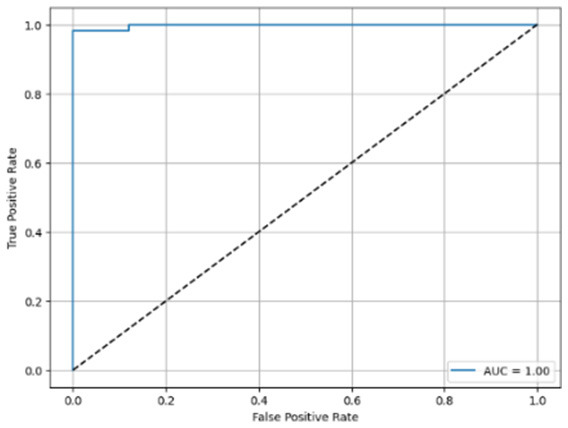	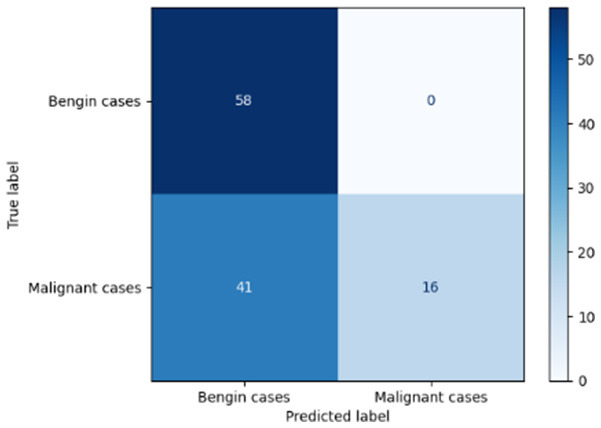	This model improves sensitivity for malignant cases and maintains high accuracy for benign ones, making it the most balanced choice. Since FN is 100%, but fails to achieve TP. Precision = 100, Recall = 0.28, F1-score = 0.43, Accuracy = 0.64
CNN five layers	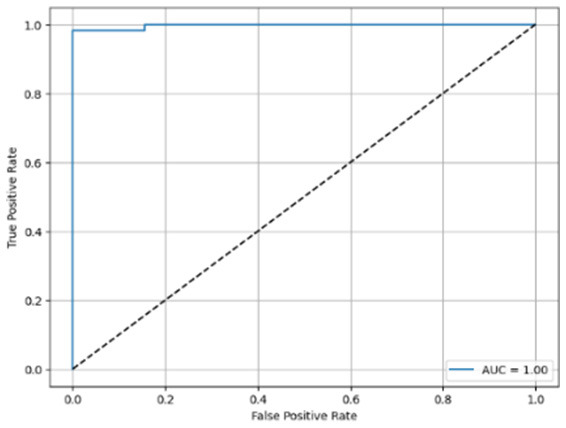	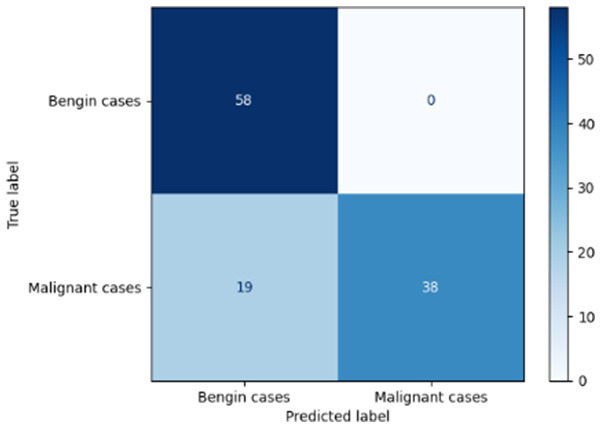	The model establishes a strong benchmark, yet models employ progressively increasing complexity. Since FN is 100%, but fails to achieve TP. **Precision** **=** **100, Recall** **=** **0.66, F1-score** **=** **0.80, Accuracy** **=** **0.83**

Bold indicates to improve the readability and highlights the values of the methods.

#### 5.2.2 Impact of PIC placement as compared to convolutional layers

Table 10 gives the results of ablation experiments looking at the variation in PIC performance with placing the controller following various numbers of convolutional layers, parameters held fixed at the optimal combination (Kp 0.6, Ki 0.05, target intensity 0.5, maximum iterations 1).

**Table 10:** Ablation Study Plot Results change the PIC based on the convolution layers (*K*_*p*_ = 0.6, *K*_*i*_ = 0.05, target intensity = 0.5, maximum iterations = 1).

Models	ROC curve	Confusion matrix	Comments and metrics
Manual PIC after three convolution layers	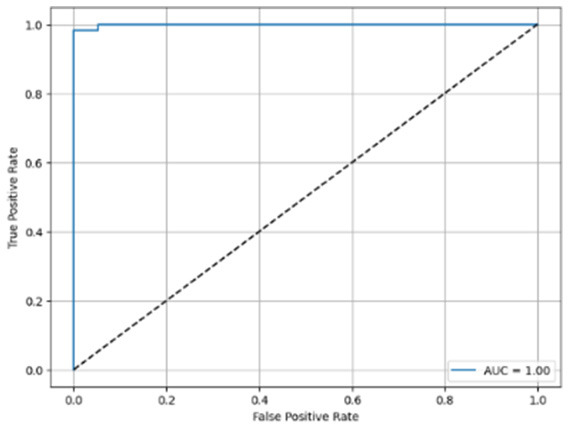	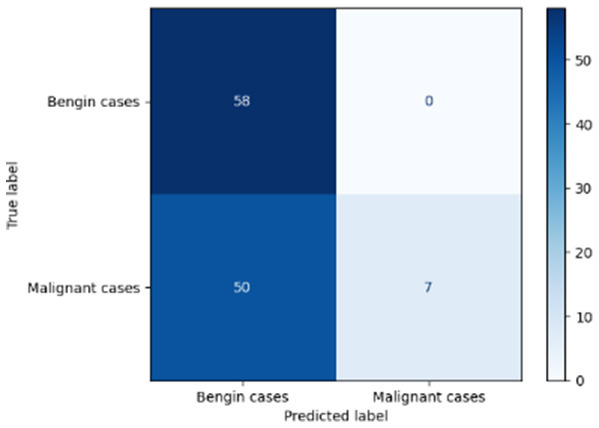	The model exhibits instability, as evidenced by its significantly low true positive values. **Precision** **=** **100, Recall** **=** **0.12,** **F1-score** **=** **0.21, Accuracy** **=** **0.56**
Manual PIC after two convolution layers	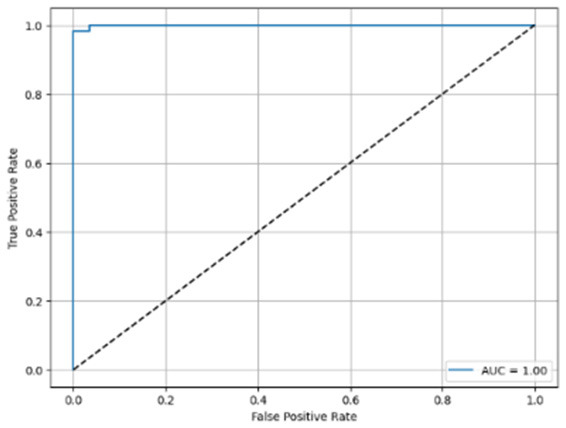	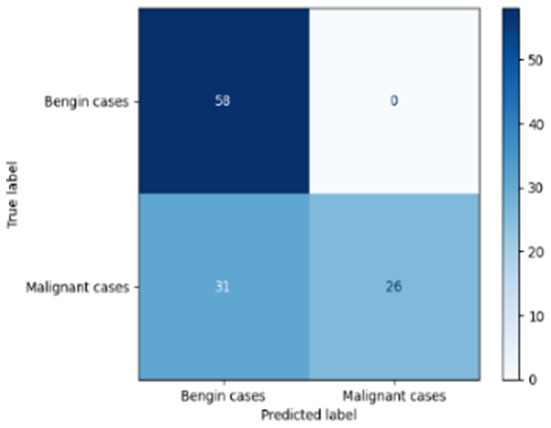	Compared to the proposed work, achieving lower accuracy results in misclassification. **Precision** **=** **100, Recall** **=** **0.45,** **F1-score** **=** **0.62, Accuracy** **=** **0.73**
Auto PIC after three convolution layers	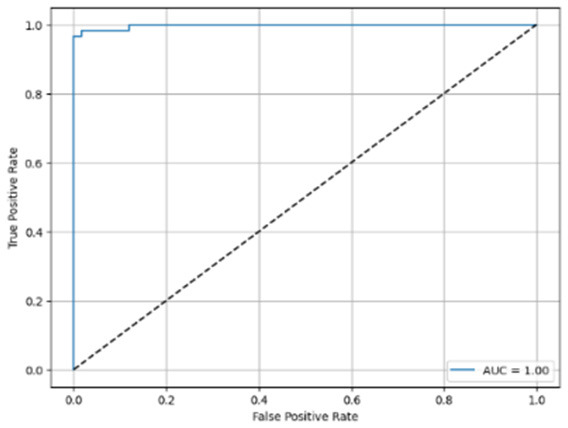	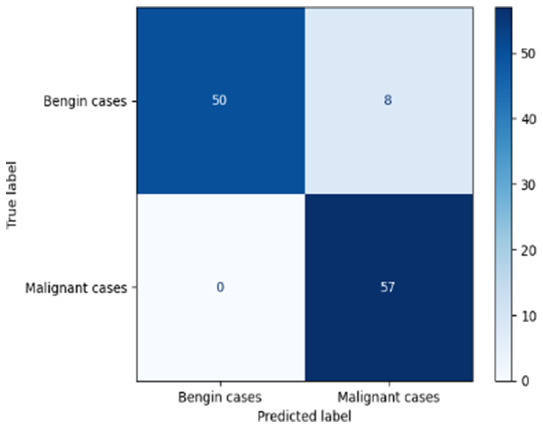	The model exhibits precise classification capabilities for the nodules; however, the performance metrics reveal certain areas necessitating enhancement. **Precision** **=** **87, Recall** **=** **1.00,** **F1-score** **=** **0.93, Accuracy** **=** **0.93**
Auto PIC after two convolution layers	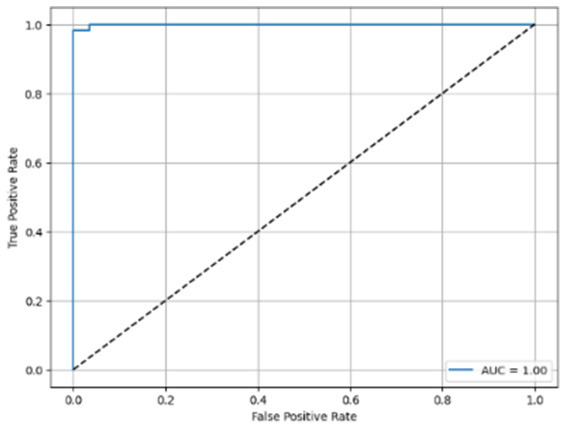	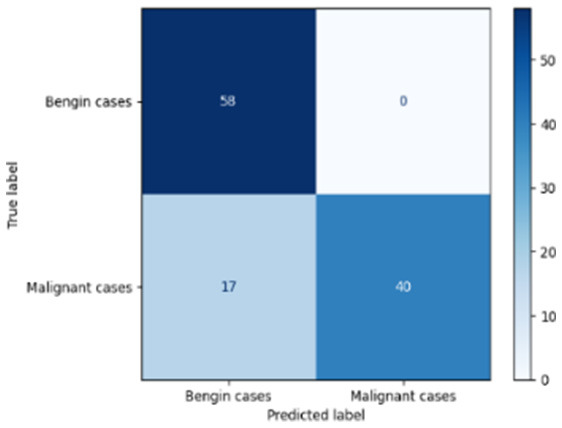	The model classifies only benign cases but performs poorly in malignancy. **Precision** **=** **1.00, Recall** **=** **0.70, F1-score** **=** **0.82, Accuracy** **=** **0.85**

Bold indicates to improve the readability and highlights the values of the methods.

The best result with any configuration tested is Precision 1.00, Recall 0.12, and F1-score 0.21 of the Manual PIC that was applied after three convolutional layers. This extreme recall deficit is due to the fact that by the third convolutional layer, the network already coded very abstract representations where the spatial boundary information that was used by the P-term is no longer available using local convolutional filtering. Application of the PIC at this phase offers practical improvement to lesion-discriminative features in addition to introducing regularization pressure, which further reduces malignant activations.

The Manual PIC, obtaining two convolutional layers, increases to Recall 0.45 and F1-score 0.62 to confirm that the previous placement of the PIC does not lose much of the spatially structured information, which the controller is expected to control. In the case of the Auto PIC in 3 convolutional layers, the performance (Precision 0.87, Recall 1.00, F1-score 0.93, Accuracy 0.93) is much higher, and learned filter adaptation partially offsets late-place detection. Nevertheless, the Auto PIC at two convolutional layers reaches Recall 0.70 and F1-score 0.82, indicating that the interaction between learned P and I filters and intermediate feature representations is non-monotonic and dependent on the level of abstraction of the feature maps at which it is inserted.

Combined, these findings support the suggested PIC placement strategy prior to the CNN or prior to the first and the second convolutional layers as the most effective ones that maintain the frequency-selective utility of the PIC mechanism.

#### 5.2.3 Impact of variable gain parameters against configurations

Table 11 is the continuation of Table 4 parameter sensitivity analysis to high gain values (Kp 1.5, Ki 0.8), as well as high iteration counts (Kp 1.0, Ki 0.05, maximum iterations 10). The high-gain setting leads to a massive loss of performance for both placement strategies: Manual PIC before-CNN has an accuracy of 0.81 and a high false positive rate, and Manual PIC mid-CNN has an accuracy of 0.68 and a recall of 0.36, a lower recall value than the CNN-only baseline. This observation is especially notable: it indicates that when improperly mis-configured, a PIC can have an active detrimental effect on the classification performance as compared to a standard CNN, accomplishing the fact that the PIC mechanism itself is not self-correcting and that principled parameter selection is required. The high iteration setting (maximum iterations 10) also worsens the performance of Manual PIC before-CNN (Accuracy 0.76, Recall 0.52), with the effect that a large number of iterations of correction errors the integral term instead of correcting them, the feature space equivalent of integrator windup. The only exception is the Manual PIC mid-CNN high iterations (Kp 0.83, Recall 1.00, F1-score 0.91, Accuracy 0.90), which indicates that the mid-CNN location is less sensitive to high iteration numbers because of the more contextual information on the middle feature maps.

**Table 11:** Ablation results of variable parameters defined for *K*_*p*_*, K*_*i*_, target intensity, and maximum iterations.

Variable parameters	Models	Confusion matrix	Metrics
*K_*p*_ =* 1.5 *K_*i*_ =* 0.8 target intensity = 0.5 max iterations = 1	Manual PIC before-CNN	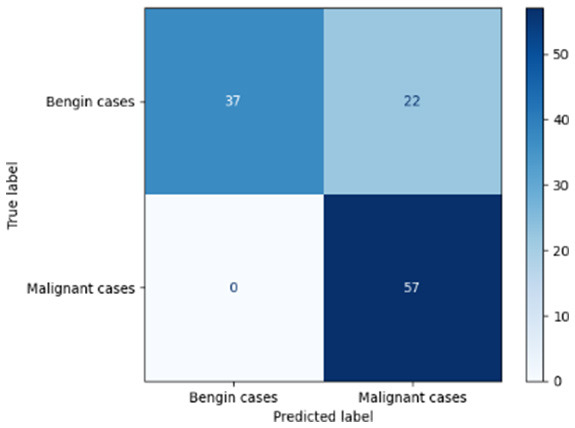	Beyond the limits of proportional and integral gains, the model shows some false positives, and its overall performance is considered inadequate. **Precision** **=** **0.72, Recall** **=** **1.00, F1-score** **=** **0.83, Accuracy** **=** **0.81**
	Manual PIC mid-CNN	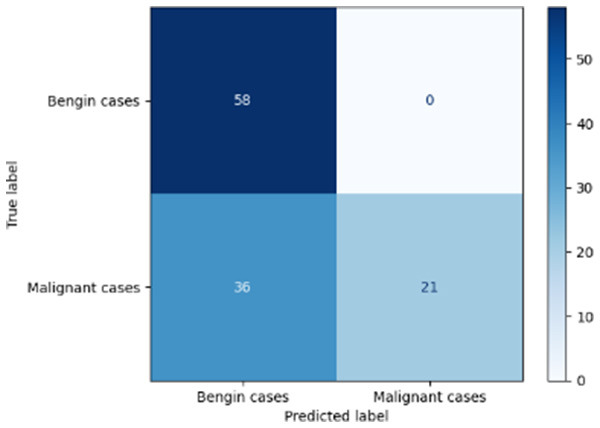	Beyond the limits of proportional and integral gains, the model exhibits a high number of false negatives. Consequently, the matrix indicates an improper class balance. **Precision** **=** **1.00, Recall** **=** **0.36, F1-score** **=** **0.53, Accuracy** **=** **0.68**
*K_*p*_ =* 1.0 *K_*i*_ =* 0.05 target intensity = 0.5 max iterations = 10	Manual PIC before-CNN	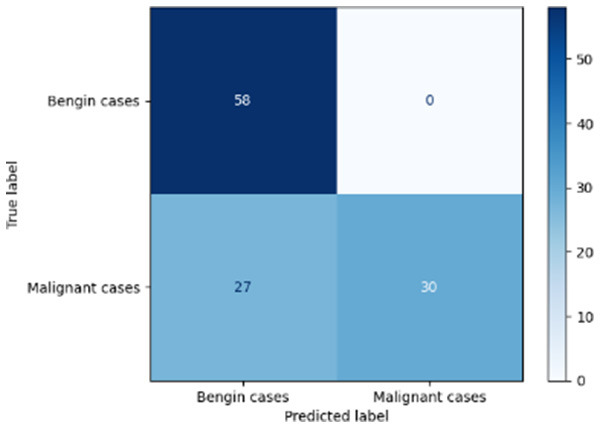	The maximum number of iterations is reached, and the model avoids false positives, so cancerous nodules are misclassified as benign. **Precision** **=** **1.00, Recall** **=** **0.52, F1-score** **=** **0.68, Accuracy** **=** **0.76**
	Manual PIC mid-CNN	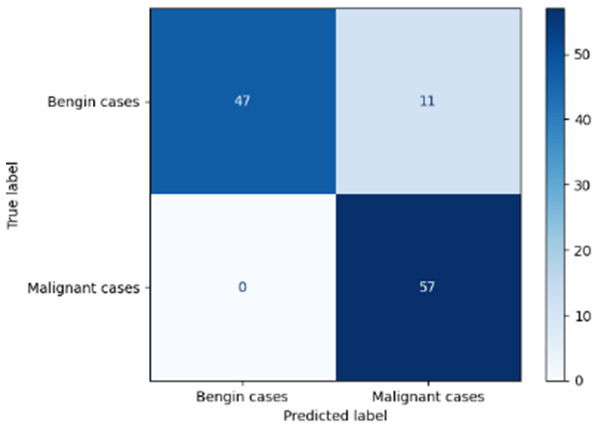	The model accurately identifies all true positives while minimizing the misclassification of benign cases, thereby reducing false positives. **Precision** **=** **0.83, Recall** **=** **1.00 F1-score** **=** **0.91, Accuracy** **=** **0.90**
*K_*p*_ =* 0.6 *K_*i*_ =* 0.5 target intensity = 0.5 max iterations = 1.0	Manual PIC mid-CNN	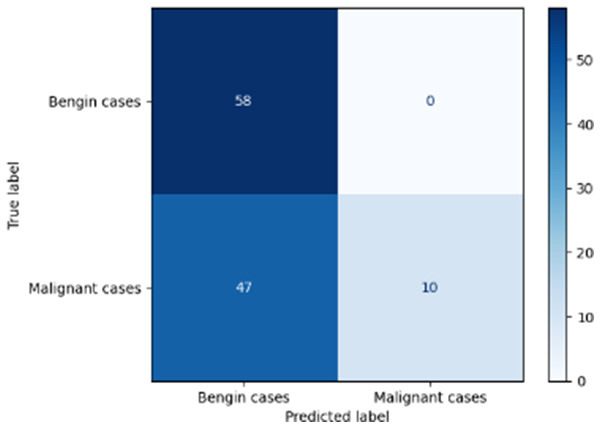	The model effectively avoids false positives in benign cases. However, it struggles to detect most malignant cases. **Precision** **=** **1.00, Recall** **=** **0.17 F1-score** **=** **0.29, Accuracy** **=** **0.59**

Bold indicates to improve the readability and highlights the values of the methods.

### 5.3 Addresses the generalization analysis

To evaluate whether the performance improvements observed in section 5.2 are attributable to the proposed architectural design rather than dataset-specific characteristics, we conducted a cross-dataset characteristics, we conducted a cross-dataset analysis using three independent lung imaging datasets, namely IQ-OTH/NCCD, LIDC-IDRI, and X-ray Lung Nodule. These datasets differ in imaging modality, acquisition conditions, annotation procedures, and lesion characteristics, thereby providing a complete assessment of model robustness as shown in Table 12.

**Table 12:** IQ-OTH/NCCD lung cancer dataset description.

Attribute	Descriptions
Full Name	Iraq-Oncology Teaching Hospital/National Center for Cancer Diseases (IQ-OTH/NCCD) Lung Cancer Dataset
Modality	Computed Tomography (CT)—DICOM format
Collection period	Three months, Fall 2019
Total CT slices	1,190 images (CT scan slices)
Total cases (patients)	110 patients
Classes	3 Classes: Malignant | Benign | Normal
Malignant cases	40 cases (malignant lung cancer)
Benign cases	15 cases
Normal cases	55 cases (healthy subjects)
Malignant images	561 CT slice images
Benign images	120 CT slice images
Normal images	416 CT slice images (original; some sources report 1,097 total slices)
CT scanner	SOMATOM (Siemens)
kVp/slice thickness	120 kV/1 mm
HU window width	350–1,200 Hounsfield Units
HU window center	50–600
Patient geography	Mainly central Iraq: Baghdad, Wasit, Diyala, Salahuddin, Babylon
Annotation	Oncologists and radiologists at Iraq-Oncology Teaching Hospital/NCCD
Image format	DICOM (.dcm), converted to JPG (512 × 512 px) for DL experiments
Class imbalance	Yes—significantly imbalanced; augmentation recommended
**Access URL (Kaggle)**	https://www.kaggle.com/datasets/hamdallak/the-iqothnccd-lung-cancer-dataset

All values represent classification accuracy. Δ indicates improvement over the baseline CNN model without PIC integration, as shown in Table 13.

**Table 13:** Cross-dataset performance comparison.

Dataset	Training	Testing	Accuracy			Δ over baseline
			CNN	PIC-before CNN	PIC-mid CNN	
IQ-OTH/NCCD-CT, 1,122 samples	906	116	0.89	0.94	0.96	+0.07
LIDC-IDRI-CT, 4,500 samples	4,054	506	0.59	0.64	0.68	+0.09
X-Ray LungNodule-Xray, 1,000 samples	800	200	0.64	0.69	0.71	+0.07

The IQ-OTH/NCCD dataset represents a relatively homogeneous imaging environment, where acquisition settings are consistent, and lesion boundaries are visually dissimilar. In this case, the starting CNN already demonstrates good performance (0.89 accuracy). The observed gain is moderate, but given that such predictions can be refined by the inclusion of past structural information, it suggests that even in situations where clear discriminative features exist, they can do so.

Stubbornly, the LIDC-IDRI data set introduces inconsistency, being significantly more varied in terms of imaging conditions, lesion appearance, and interrater consistency. This decrease in the baseline performance (0.59 accuracy) indicates the challenge posed by this dataset. Both PIC-based models have a particularly strong advantage, with PIC-mid having an accuracy of 0.68 and the greatest relative improvement (+0.09). This suggests that prior information integration contributes to stabilizing feature learning when using heterogeneous data and less consistent annotations.

The X-Ray Lung Nodule dataset also poses a challenge to the model because the imaging modality is different. In contrast to CT scanning, X-Ray images include overlapping structures of the anatomy with poor contrast and may blur the boundaries of the lesion. As a result, the baseline CNN achieves lower performance (0.64 accuracy). The inclusion of PIC improves performance to 0.71(PIC-mid), demonstrating that the proposed approach remains beneficial even under modality variation. However, the overall performance remains lower than that observed for CT datasets, indicating that modality- specific differences still impact generalization.

Across all datasets, PIC-mid consistently outperforms PIC-before by nearly 2–4% points. The mid-level integration allows early convolutional layers to learn modality-specific low-level features without constraint, while integrating structural priors at deeper stages where spatial and semantic relationships become more meaningful. In contrast, applying PIC at the input stage may restrict early feature extraction, particularly when data distributions vary across datasets.

Overall, the results demonstrate that the proposed PIC integration provides consistent performance improvements across datasets with varying characteristics. This supports the arguments that the observed gains are linked to the architectural design rather than being limited to a specific dataset. At the same time, reduced performance is more on heterogeneous and cross-modality datasets. This highlights that generalization remains influenced by data variability. Therefore, PIC enhances robustness; additional strategies, such as domain adaptation or dataset fine-tuning, may be required. This is necessary for deployment in diverse clinical settings. These results reinforce the importance of evaluating deep learning models on multiple independent datasets to ensure stability, practical applicability, and reliability in real-world settings.

### 5.4 Limitations

#### 5.4.1 Computational overhead

A standard CNN has only one trainable parameter compared to a full PIC setting, which has two learned convolutional filter kernels (P and I paths) in each PIC block with dimensions C x k^2^, where C is the number of channels and k is the size of the kernel. Although this overhead is small in comparison to the resolutions and batch sizes considered in this study, it can pose practical limitations in resource-constrained clinical implementation environments like edge devices or real-time intraoperative imaging systems. Future studies may explore parameter-efficient implementations of PIC, such as depthwise separable filter designs (Howard et al., 2017), to reduce the computational cost less but maintain frequency-selective control of features.

#### 5.4.2 Manual parameter calibration load

In the PIC setup, four parameters are to be chosen, namely, *K*_*p*_, *K*_*i*_, desired intensity, and maximum iterations by searching validation sets. These values are very sensitive to performance, as seen by Tables 7, 8, and inappropriate settings may worsen performance to a lower level than the CNN-only baseline. This provides a practical barrier of accessibility to clinical practitioners without control-theoretic knowledge. Although the auto PIC averts this by learning filter parameters by backpropagation, it becomes hypersensitive to learning rate and weight initialization. Future efforts should investigate automated gain scheduling schemes or Bayesian hyperparameter optimization in order to alleviate the tuning complexity in both schemes.

#### 5.4.3 Generalization scope

The improvements in performance that were shown in this experiment were achieved on datasets of small size (1,000 to 4,500 samples) and only in binary classification of lung nodules. It has not been tested on generalization to the multi-class nodule subtype classification to other anatomical locations (e.g., breast, colon, brain) or other imaging modalities other than CT and X-ray. The lower performance of the LIDC-IDRI and the X-ray dataset is an indication that the PIC mechanisms frequency-selective filters have been implicitly trained to the imaging properties of the primary training dataset, constraining out-of-domain robustness. The future research can take the directions of domain adaptation strategies or modality-specific PIC filter initialization to extend these limitations.

## 6 Conclusion

The integration of a Proportional Integral Controller (PIC) into the CNN architecture significantly improves dynamic learning behavior and classification accuracy in medical image analysis. Two methodologies were explored: manual and automated control implemented in two distinct configurations: PIC before CNN and PIC mid CNN. In the PIC before CNN arrangement, the controller operates as a preprocessing layer, normalizing grayscale intensity values to the desired target range. This early stabilization enables the CNN to learn from a regulated feature representation, reducing fluctuations during early learning stages. In the manual PIC configuration, controller parameters are predefined based on expert knowledge, allowing fine-grained control over feature normalization and improving accuracy under controlled experimental conditions. In contrast, the automated PIC before CNN dynamically adjusts control parameters based on image characteristics, resulting in improved robustness and consistency across heterogeneous datasets.

The scheme PIC mid-CNN inserts the control mechanism directly into the mid layers, giving the possibility of the direct lapse of the internal learning representations. This combination makes the relationship with the CNN learning process better, which positively affects the feature abstraction and accelerates the convergence rate. Nonetheless, empirical results have shown that the Automated PIC mid-CNN has the best classification accuracy and robustness, which confirms that the adaptive control systems embedded in learning layers achieve a better performance. The combination of the control theory and CNNs introduces a convenient methodology for integrating fusion in the classification of medical images. The method does not require hyperparameter optimization and does not need dense and flattened layers, which provides a simple solution to the analysis of medical image classification. Medical image dataset results of experiments demonstrating the integration of PIC with CNN highly improves accuracy of classifications, generalization, and interpretability, particularly in challenging conditions where images are of low quality or noisy ones.

### 6.1 Drawbacks

The observed performance primarily reflects the LIDC-IDRI datasets characteristic clinical complexity and variability, not a vigorous model flaw. This aligns with its established role as a severe benchmark, a key limitation in generalizing to highly heterogeneous real-world data.

### 6.2 Future work

The performance on different data and populations, e.g., from MRI and PET scans should be evaluated to test the PIC-CNN for broad applicability. It is important that the PIC-enhanced CNN runs fast enough for use in a hospital setting, while not sacrificing accuracy. We may improve this model's image classification results by incorporating or feeding attentional mechanisms with other models.

## Data Availability

The original contributions presented in the study are included in the article/Supplementary material, further inquiries can be directed to the corresponding author.

## Footnotes

1
https://dagshub.com/glossary/internal-covariate-shift/

2
https://medium.com/@abhishekkumargupta5557/what-is-internal-covariate-shift-e682c026c29b
